# Reactions of Surface Peroxides Contribute to Rates and Selectivities for C_2_H_4_ Epoxidation on Silver

**DOI:** 10.1021/acscatal.4c06945

**Published:** 2025-01-09

**Authors:** Ching-Tien Chen, Anna Sviripa, Sugandha Verma, Christopher Paolucci, David W. Flaherty

**Affiliations:** † School of Chemical and Biomolecular Engineering, Georgia Institute of Technology, Atlanta, Georgia 30332, United States; ‡ Department of Chemical Engineering, University of Virginia, Charlottesville, Virginia 22904, United States

**Keywords:** ethylene epoxidation, unpromoted silver nanoparticles, operando Raman spectroscopy, kinetic measurements, DFT calculations

## Abstract

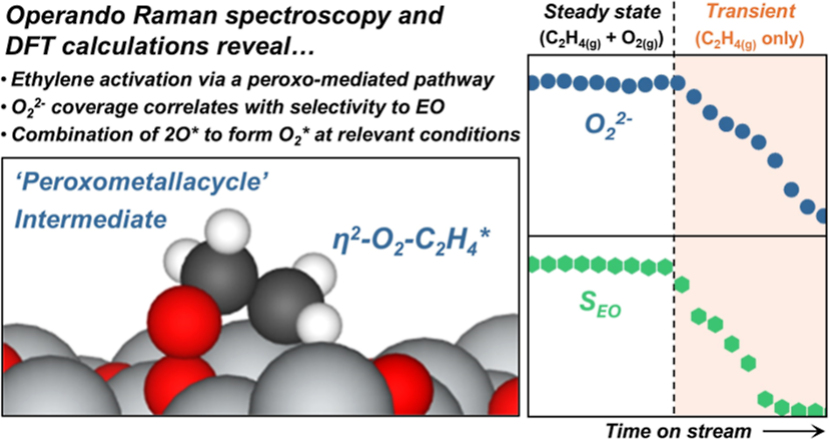

Partial oxidation of ethylene over silver catalysts produces more than 30 million metric tons of ethylene oxide (EO) annually. However, the form of active silver surfaces, reactive oxygen species, and dominant pathways of this chemical reaction remains controversial despite decades of research. Here, we use *operando* Raman spectroscopy and transient kinetic measurements to demonstrate that higher coverages of peroxide species, present only upon Ag oxide surfaces that form *in situ*, correlate with greater selectivities to EO. *Ab initio* calculations reveal that the reconstructed Ag oxides preferentially stabilize diatomic oxygen species (peroxide and superoxide) under relevant conditions, and these species contribute to the selective formation of EO. The dominant reaction pathways change with surface coverages; however, bound O_2_ consistently activates by reaction with C_2_H_4_, and products form subsequently through peroxo- and oxometallacycle surface complexes. Taken together, density functional theory calculations and kinetic and transient experimental measurements show that the formation of peroxide intermediates requires oxidation of the Ag surface (via subsurface oxygen), and an increase in surface peroxides coincides with the highest EO selectivity for the unpromoted Ag catalyst. These findings suggest that the promoters ubiquitous for ethylene epoxidation (e.g., chlorine, transition metals, and alkali metals) may succeed by oxidation of Ag and increasing coverages of peroxides at industrial conditions.

## 1 Introduction

Production of ethylene oxide (EO) over silver nanoparticles is the largest-scale catalytic partial oxidation process in industry, producing over 30 million metric tons (MMt) of EO worldwide in 2021.^1,2^ This remarkable scale cannot be achieved without promoters (e.g., trace amount of chlorine and transition metals), which push EO selectivity to over 85% against the formation of carbon dioxide (CO_2_).^2,3^ Despite the importance of this chemistry, there is no solid agreement about the mechanisms of promoter effects, which hinders the technical development to further improve the selectivity and reduce the intense CO_2_ emissions from this industry (e.g., 6.7–15 MMt/yr just by considering the reaction stoichiometry at an EO selectivity of 80–90%^1,4^). The structure of the catalytic surface, the dominant reaction pathways, and the nature of the reactive oxygen species that form EO or CO_2_ remain controversial even for unpromoted silver nanoparticles after half a century of investigations.^4^

Descriptions for steady-state reaction rates were established at relevant conditions; however, the surface species formed on silver catalysts were mainly examined using silver single crystals, *ex situ* or with surface science tools at extremely low pressures.^5−7^ These investigations suggested that electrophilic oxygen intermediates [O(1s) binding energy near 530 eV] facilitate the selective formation of EO and that CO_2_ forms rapidly following kinetically relevant H atom abstraction from a C_2_-intermediate. Foundational reports by Linic and Barteau revealed a surface oxametallacycle (OMC) formed by the ring-opening of the adsorbed EO onto the surface of Ag(111) single crystal at ∼250 K and ultrahigh vacuum conditions, which reformed EO or dissociated to ethylene as the temperature increased.^8−10^ This OMC species was considered a key intermediate for the reactions between atomic oxygen (O*) and C_2_H_4_ by employing the principle of microscopic reversibility and density functional theory (DFT) calculations.^11^ Consequently, promoters were hypothesized to stabilize the transition state for EO formation by changing the electronic structure of the OMC intermediates.^12^ In contrast, Özbek and van Santen examined reactions on Ag_2_O(001) with DFT calculations and suggested that surfaces with high oxygen coverages mediate a direct epoxidation reaction between C_2_H_4_ and O*, forming primarily EO and bypassing the less EO-selective pathways involving the formation of OMC.^13−15^ Following this observation, van Santen et al. suggested that chlorine promoters occupy oxygen vacancies on Ag surfaces to reduce the coverages of OMC.^16^ Many of these formative investigations involved crystalline models and ultrahigh vacuum experiments, which differ sharply in coverage and structure from the catalysts with high surface coverages that provide steady-state rates. To affirm the role of promoters, the structure of Ag, reactive intermediates, and formation mechanisms of EO first need to be confirmed under relevant conditions.

Recent reports provide strong evidence (e.g., *ab initio* phase diagrams, electron microscopy) that surfaces of Ag catalysts reconstruct to form a partially oxidized layer (i.e., stoichiometry Ag_2_O_≤1_) at temperatures and chemical potentials of oxygen relevant to industrial EO catalysis.^17,18^ In our previous work, we demonstrated that reconstructed Ag_2_O_≤1_ surfaces bind high coverages of O* but also O_2_* and C_2_H_4_* at catalytic conditions using *in situ* Raman spectroscopy and DFT calculations.^19^ These findings were affirmed recently by Wachs et al. through isotope labeling and further computation,^20,21^ and the surface O_2_* was proposed to adopt a Ag_4_–O_2_* structure. Features that signify the Ag_4_–O_2_* attenuated when the Ag catalysts contacted C_2_H_4_ and the catalyst temperature increased from ambient to 573 K. The selectivity to EO simultaneously decreased while the temperature increased. These experiments and recent Raman vibrational assignments aided by DFT calculations hint that surface O_2_* species possess a role in ethylene epoxidation; however, the observed surface changes and kinetics may also reflect restructuring of the Ag oxide surface, the simple dissociation of O_2_* (without reaction with C_2_H_4_), or incommensurate changes in apparent rate constants for the competing reaction pathways induced by the temperature differences. Distinguishing among these possibilities experimentally requires direct correlation between structural characterization of reactive surfaces, determination of coverages for diatomic (O_2_*) and monatomic (O*) oxygen species, and evaluation of ethylene epoxidation rates and selectivities.

Here, we link both instantaneous and steady-state assessment of the catalytic surface to rates and selectivities at different epoxidation conditions by time-resolved *operando* Raman spectroscopy, which reveals the distinct reactivity of different O_2_* and monatomic O* on Ag surfaces. Additionally, we give evidence from combinations of DFT, kinetic, and spectroscopic measurements for mechanisms that describe activation of O_2_* by reaction with C_2_H_4_ and the separate pathways that O_2_* and O* species traverse to form EO and CO_2_ upon these oxidized Ag surfaces. These observations indicate that EO selectivities depend directly upon the surface coverage of O_2_*, which provides additional insight useful in explanations for the effects of common promoters.

## 2 Results and Discussion

**Figure 1:**
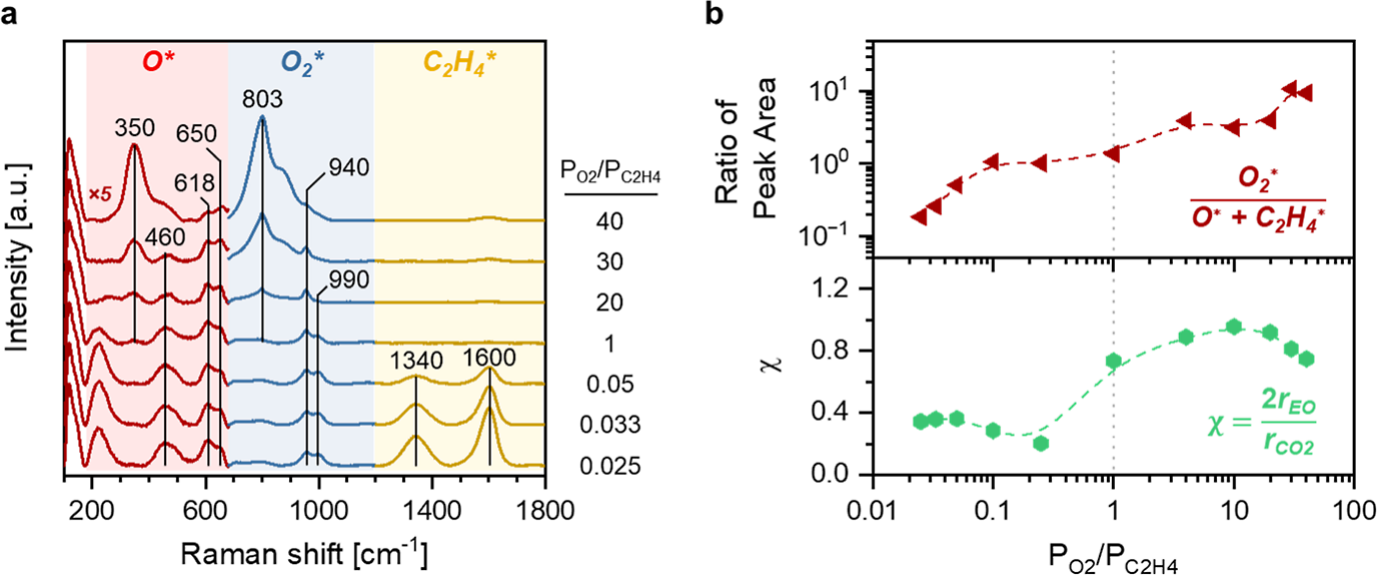
*Operando* Raman spectroscopy obtained at steady-state across ratios of partial pressures of O_2_ to C_2_H_4_ at 523 K. (a) Representative Raman spectra of the Ag surface and surface intermediates, and (b) ratio of peak area of diatomic oxygen to other surface species and ratios of the EO to CO_2_ formation rates (defined as χ = 2·*r*_EO_/*r*_CO_2__) as functions of the ratios of O_2_ to C_2_H_4_ partial pressures (*P*_O_2__/*P*_C_2_H_4__, 2–80 kPa O_2_, 2–80 kPa C_2_H_4_). Catalyst: unsupported silver nanoparticles. All Raman spectra were normalized by the area of peak in the range of 100–200 cm^–1^.

We began by obtaining Raman spectra during steady-state ethylene epoxidation upon unsupported Ag nanoparticles [selected to avoid the convolution of intermediates present upon an oxide support (Figure 1a)], where we observed that the Ag surface binds a variety of oxygen- and ethylene-derived species with varying coverages across a 1600-fold span of *P*_O_2__/*P*_C_2_H_4__ ratios. Following each change in *P*_O_2__/*P*_C_2_H_4__, steady-state rates and surface coverages were achieved only after at least 24 h. Previously, rates and selectivities for ethylene epoxidation on supported silver catalysts with and without promoters were known to vary over periods of many hours,^22−24^ but here, *in situ* Raman demonstrates that these transients reflect changes in the surface structure. The evolution of *in situ* Raman spectra and product formation rates following a step change in reactant pressures (Figure S2) affirm more specifically that these long periods of transient behavior signify the redistribution of oxygen species between the surface and subsurface regions.^24^

**Scheme 1:**
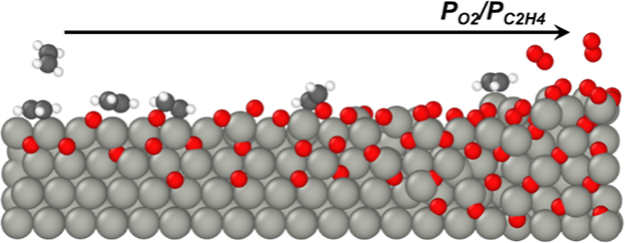
Depiction of Changes to the Surface of the Silver Catalyst across the Span of Epoxidation Conditions, Where Spheres Represent Silver (Light Gray), Oxygen (Red), Carbon (Dark Gray), and Hydrogen (White) Atoms

We assigned Raman features between 200 and 650 cm^–1^ to several structures that involve surface and subsurface O*, features between 700 and 1200 cm^–1^ to *v*(O–O) vibration of surface O_2_*, and peaks centered at 1340 and 1600 cm^–1^ to the δ(CH_2_) and *v*(C–C) modes of adsorbed ethylene (C_2_H_4_*), which agrees with the vibrational frequencies calculated using DFT in recent studies.^19−21^ The broad and numerous Raman features for both O* and O_2_* species reflect diverse local environments and electron densities for these oxygen species, consistent with implications of near-ambient pressure (25 Pa) measurements with X-ray photon spectroscopy (XPS).^25,26^ The O_2_* species at 803 cm^–1^ possesses greater charge transfer from silver and a longer O–O bond distance compared to the O_2_* species at 940–990 cm^–1^. Consequently, we propose that O_2_* at 803 cm^–1^ resembles a peroxo (O_2_^2–^) species, while those at 940–990 cm^–1^ represent superoxo (O_2_^–^) species.^27,28^ While coverages of oxygen appear greatest at the highest ratios of *P*_O_2__/*P*_C_2_H_4__, the surface retains significant coverages of the O* and the O_2_* species even at the most reducing conditions (*P*_O_2__/*P*_C_2_H_4__ → 0.025). Changes in the areas of these features reflect relative changes in the coverages of these species (depicted in Scheme 1); however, absolute coverages cannot be determined since extinction coefficients remain unknown.

We then correlated the relative peak areas of surface species obtained by *operando* Raman spectroscopy to the selectivities to EO (Figures 1b and S4) expressed as χ, which equals the ratio of EO to CO_2_ formation rates per C_2_H_4_ molecule consumed (χ = 2·*r*_EO_/*r*_CO_2__). Values of χ increase as ratios of *P*_O_2__ to *P*_C_2_H_4__ rise from 0.1 to 10, which coincides with the number of O_2_* compared to other surface species (i.e., combined coverage of O* and C_2_H_4_*). The increases in EO selectivity with increasing *P*_O_2__ contradict predictions from the microkinetic model proposed by Setiawan et al.,^29^ which indicates that the single reaction between C_2_H_4_ and O_2_* cannot describe experimental selectivity trends that also reflect contributions of other surface oxygen species. In summary, the greatest values of χ appear for Ag surfaces with larger overall coverages of diatomic oxygen relative to C_2_H_4_-derived intermediates, which suggests that both the coverage of O_2_* and the kinetics of EO depend strongly upon the extent of oxidation of the silver surface.

**Figure 2:**
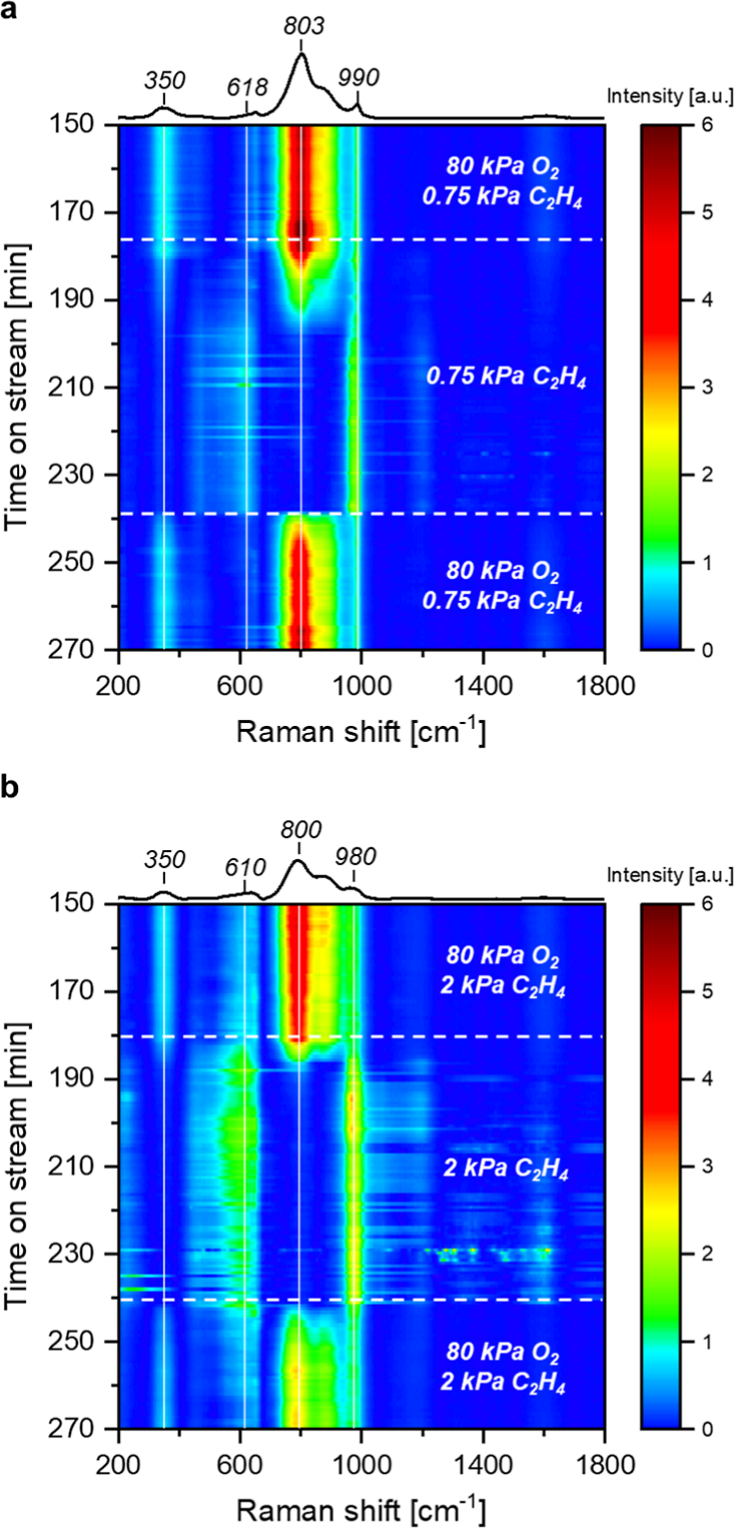
Time-resolved Raman spectra obtained by achieving steady-state rates, selectivities, and coverage (*t* ≤ 180 min), subsequently removing O_2_ from the reactant stream while continuing flow of C_2_H_4_ in order to consume reactive forms of surface oxygen and returning to a combination of both O_2_ and C_2_H_4_ (*t* ≥ 240 min) with (a) 0.75 kPa C_2_H_4_ and (b) 2 kPa C_2_H_4_, both with 80 kPa O_2_ at 523 K. Spectra at the top of the plots represents the surface observed at steady-state, and the colors signify Raman scattering intensity, with the greatest intensities in red and the lowest intensities in blue. Catalyst: unsupported silver nanoparticles. All Raman spectra were normalized by the area of the peak in the range of 100–200 cm^–1^.

**Figure 3:**
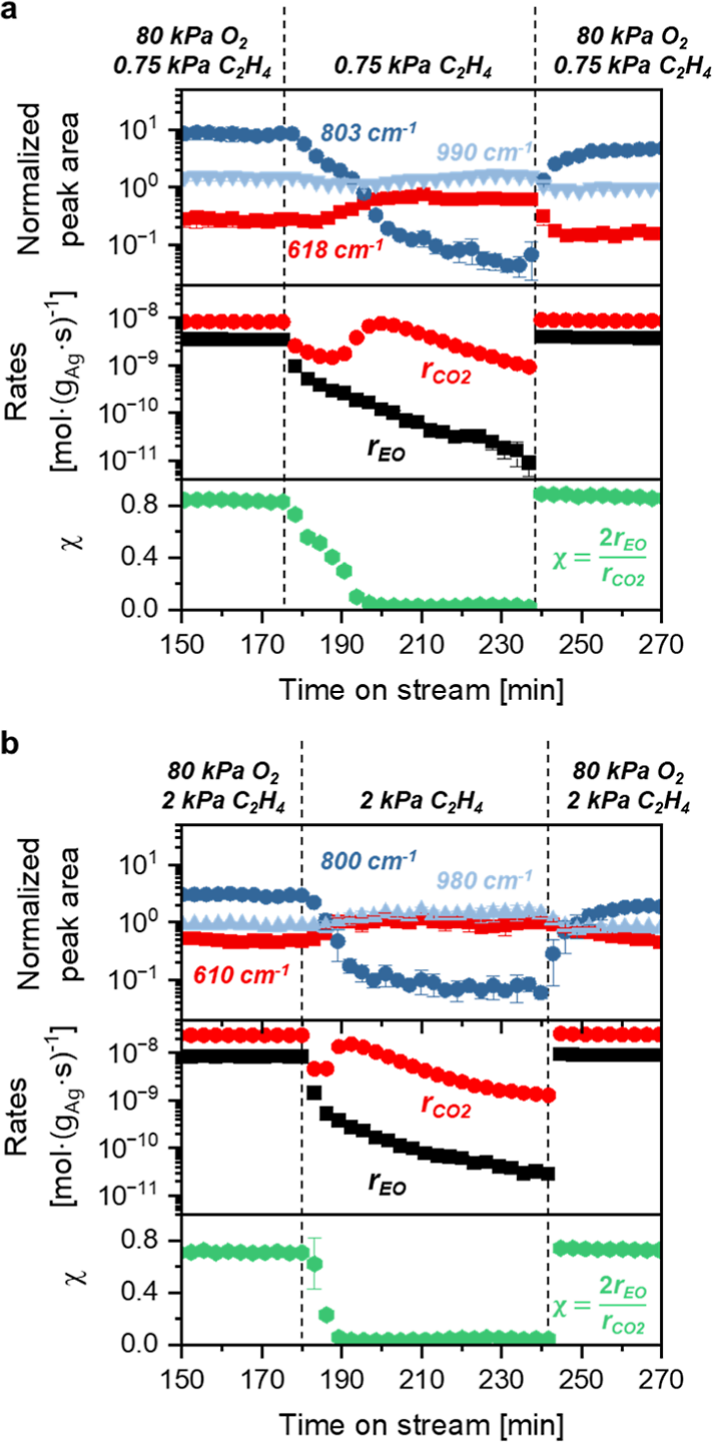
Normalized peak areas of Raman features (adapted from Figure 2), instantaneous formation rates of EO and CO_2_, and values of χ as functions of time during experiments in which the system achieved steady-state (*t* ≤ 180 min) and O_2_ was removed while flowing C_2_H_4_, followed by reintroduction of both O_2_ and C_2_H_4_ (*t* ≥ 240 min) at (a) 0.75 kPa C_2_H_4_ and (b) 2 kPa C_2_H_4_, both with 80 kPa O_2_ at 523 K. Data points represent an average of three repeated transient experiments, with error bars showing a single standard deviation. Catalyst: unsupported silver nanoparticles. All Raman spectra were normalized by the area of the peak in the range of 100–200 cm^–1^.

We conducted a series of transient *operando* experiments that relate the spectrokinetics of surface intermediates on Ag surfaces with high O_2_*-coverages to instantaneous formation rates of gaseous EO and CO_2_. Figures 2a and 3a show one instance where the system achieved steady-state rates and Raman spectra (80 kPa O_2_, 0.75 kPa C_2_H_4_, 523 K), after which, the O_2_ flow ceased and only dilute ethylene (0.75 kPa C_2_H_4_, balance He) continued to flow. Following the cessation of flow of the O_2_ into the reactor, formation rates of EO and normalized Raman peak areas for the O_2_^2–^ (803 cm^–1^) decrease exponentially. Notably, the area of the peaks for O_2_^–^ (990 cm^–1^) remains nearly constant throughout the experiment. In contrast to the monotonic decreases in EO rates, the formation rate of CO_2_ decreases initially before increasing by 10-fold, coincident with an increase in the O* peak area (618 cm^–1^). Analogous transient experiments performed at greater partial pressures of C_2_H_4_ (2 kPa C_2_H_4_, Figures 2b and 3b; 1 kPa C_2_H_4_, Figure S6) give comparable results but show more rapid attenuation of Raman features for O_2_^2–^, which reflects greater rates of consumption of O_2_^2–^ by reactions with ethylene. The distinct correlations between EO rates and O_2_^2–^ features taken together with those between CO_2_ rates and O* peaks suggest that EO forms by reactions with O_2_^2–^ and CO_2_ forms by processes that involve O* species. The extinction coefficient at 618 cm^–1^ for O* depends on O_2_^2–^ coverage;^19^ therefore, the greater peak area at 618 cm^–1^ does not necessarily reflect a proportional increase in O* coverage. Nevertheless, these comparisons evince a decrease in EO selectivities with decreasing coverage of O_2_^2–^ in both steady-state (Figure 1) and transient experiments (Figures 3 and S6) and a decrease in EO rates when surface coverages of O_2_^2–^ decrease. Coverages of reactive intermediates remain constant when pure helium replaces the reaction mixture (Figure S9), which demonstrates that the decrease in O_2_^2–^ coverage during contact with flowing ethylene (Figures 2 and 3) represents consumption by reactions with ethylene and not by the desorption of O* or O_2_* species as O_2_.

The cumulative amount of EO and CO_2_ (and H_2_O) formed during the transient period (Figure 3, 180–240 min) depends linearly upon the total coverage of all reactive forms of oxygen. Based on the stoichiometry of these reactions, the quantity of reactive oxygen species corresponds to ∼36 μmol O_atom_ g_Ag_^–1^, which corresponds to a 1–2 nm film of reconstructed Ag_2_O on the external surface of the silver nanoparticles (80–100 nm diameters, Section S2.3 and Figure S8).^21^ These calculations reconcile the different conclusions reached in studies that utilize distinct characterization methods. For example, bulk characterization methods previously employed (e.g., X-ray diffraction) suggest that the nanoparticles remain metallic during ethylene epoxidation,^24^ whereas surface-sensitive methods (e.g., X-ray photoelectron spectroscopy^30^ and Raman spectroscopy^19,21^) demonstrate the presence of high oxygen coverages and surface reconstructions.

Taken together, observations from these *operando* transient experiments suggest that O_2_^2–^ and O* react with C_2_H_4_ to produce EO and CO_2_, and each form of oxygen possesses a different intrinsic selectivity. We postulate that EO can form through an O_2_*-assisted C_2_H_4_ activation pathway (i.e., C_2_H_4_ + O_2_* → EO + O*). This process consumes primarily O_2_^2–^, as opposed to O_2_^–^ and O*, and simultaneously produces one O* atom per O_2_^2–^ depleted. Consequently, decreasing O_2_^2–^ results in increasing O* coverages, which facilitates the formation of CO_2_ and reduces the values of χ. Campbell and co-workers made a similar proposal decades earlier, inspired by the detection of chemisorbed diatomic oxygen on Ag single crystals at cryogenic temperatures (*T* < 200 K).^31−34^ The mechanism proposed by Campbell assumed irreversible dissociation of diatomic oxygen, which leads to maximum selectivity toward EO equal to 85.7% (i.e., 6/7), based on the idea that O_2_* reacts with ethylene to form EO and the residual O* only produces CO_2_ and H_2_O.^35^ This hypothesis, however, was refuted when further catalyst development resulted in processes that give EO selectivities in excess of 90%.^36^ Contemporaneously, Grant and Lambert argued that only chemisorbed atomic oxygen reacts with C_2_H_4_, based on temperature-programmed reaction experiments at low oxygen pressure (∼300 Pa) followed by surface characterization in a ultrahigh vacuum chamber.^37^ The community subsequently discounted direct reactions involving O_2_* and converged on the conclusion that O* reacts with ethylene to form both EO and CO_2_, while O_2_* serves as a pool of O* through dissociative adsorption.^38,39^ Recently, Pu et al. performed a temperature-programmed experiment (from 298 to 473 K) by flowing C^18^O_2(g)_ to a silver surface preoxidized with ^16^O_2(g)_, showing that oxygen species exchange to produce ^18^O_2_*, C^16^O_2(g)_, and C^16^O^18^O_(g)_, which suggests that O_2_* on Ag surfaces may be involved in surface reactions. Here, we provide experimental evidence that O_2_^2–^ and ethylene react directly (Figures 1 and 3). We next sought atomistic insight into how these reactions proceed on the oxidized Ag surface present under representative reaction conditions.

**Figure 4:**
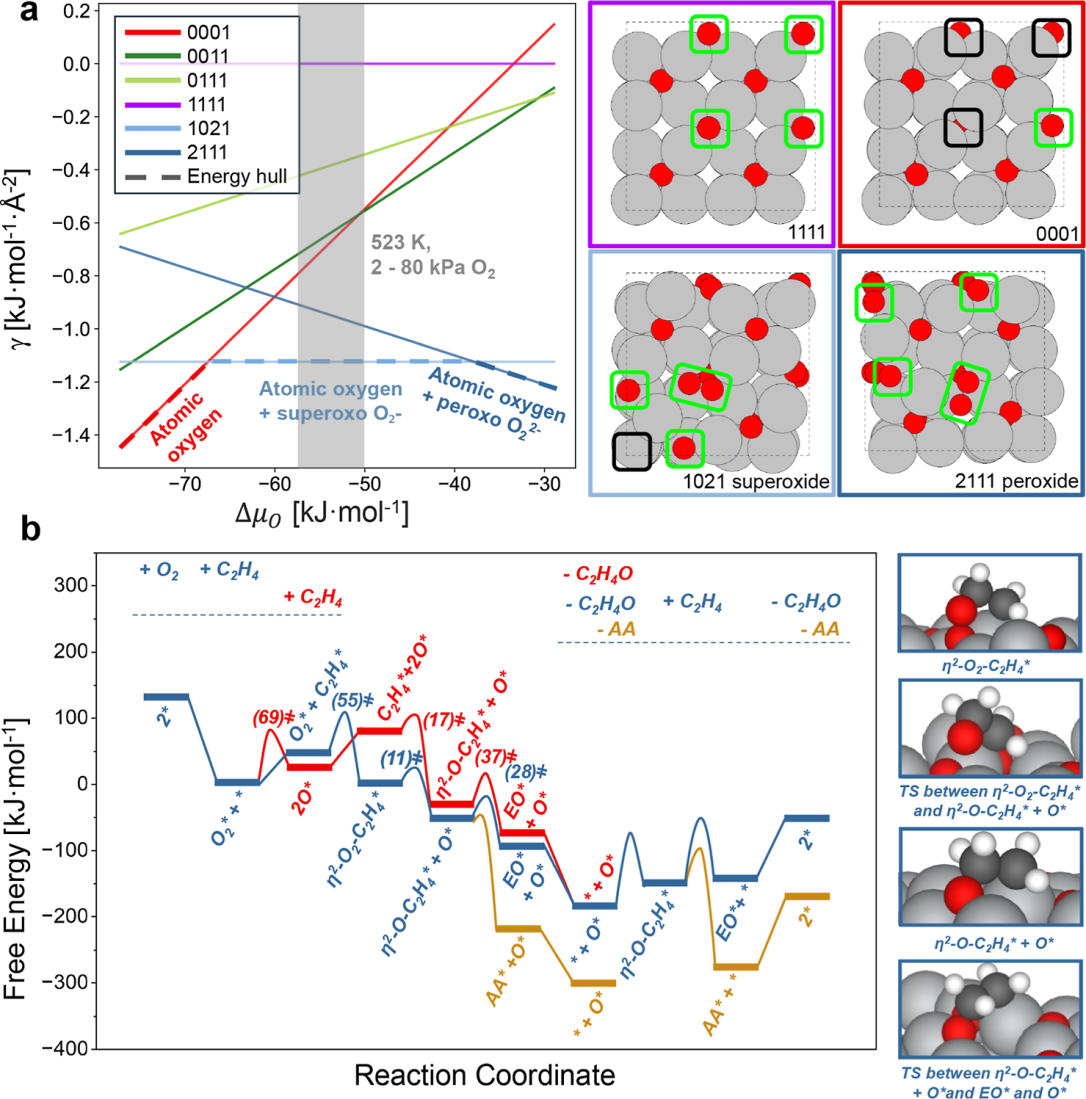
(a) Free energy diagram for surfaces with different oxygen species. Shaded region represents experimental conditions (Δμ_O_ = −58 to −50 kJ mol^–1^ correspond to 523 K and 2–80 kPa O_2_). Surfaces are labeled with four integers that represent different species adsorbed on the four adsorption sites, where “0” is O_vac_, “1” is O*, and “2” is O_2_*. Bright green boxes—surface O* or O_2_*, black boxes—O_vac_. Atom colors: O—red, Ag—light gray, C—dark gray, H—white. Black dashed line represents the cell boundary. (b) Free energy coordinate of reactions among C_2_H_4_ and O_2_ derived intermediates upon the Ag oxide surface. Values in parentheses indicate intrinsic activation free energies. Free energies were calculated at 523 K, 80 kPa O_2_, 0.75 kPa C_2_H_4_, 0.15 kPa EO, and 0.0375 kPa AA. Molecular formulas along the top of the diagram indicate adsorption (+) and desorption (−) of gaseous species.

We examined reaction pathways among C_2_H_4_- and O_2_-derived intermediates using representative molecular models for oxidized Ag surfaces,^24,40^ which was motivated by the knowledge that the Ag surface oxidizes and reconstructs within reactive environments and our interpretation of the *operando* spectroscopy and transient experiments (*vide supra*). Starting from the Ag_2_O surface used by van Santen and co-workers^13,15^ (Section S4.2 contains further details), Figure 4a depicts ensembles of symmetrically distinct models that contain combinations of surface O vacancies (O_vac_) and O_2_*. The most thermodynamically stable surfaces at relevant oxygen chemical potentials contain O_2_* species with O_2_^2–^ (peroxo) present on the oxygen-saturated surface (no vacancies at the surface) and O_2_^–^ (superoxo) dominant on the surface with an oxygen vacancy. The speciation of diatomic forms of oxygen was derived from Bader charge analysis and calculated vibrational frequencies (Figure S3 and Tables 1 and S4 and S5). The lowest free energy of peroxo and superoxo structures possesses vibrational frequencies of 931 and 1155 cm^–1^, respectively, in comparison to the experimental values of 803 and 940–990 cm^–1^ and are shifted ∼200 cm^–1^ apart and match a comparable shift between the observed peaks in the experimental spectra assigned to these species. Previous computed vibrational frequencies of O_2_* species on Ag_2_O surfaces span the range 800–1100 cm^–1^ and depend on the exact configuration of the surface,^19^ which shows that the frequencies strongly sense the choice of the molecular model. Consequently, the differences between the computed and experimental frequencies here likely reflect deviations between the Ag_2_O model and the more complex nature of the oxidized and reconstructed Ag_*x*_O_*y*_ surfaces present during catalysis. Nevertheless, the computed structures evidence distinct oxygen species on an oxidized Ag surface and provide a useful model to examine the catalytic contributions of each form of oxygen. Thermodynamic and Bader charge analyses (Figures 4a and S3) suggest that electron transfer from Ag to O_2_* increases as the surface becomes more oxidized, which agrees with the expectation that molecular oxygen binds more exothermically to Ag surfaces that contain subsurface oxygen as reported by Mavrikakis et al.^41^ In contrast, formation of O_2_* on metallic Ag or the *p*(4 × 4) reconstruction are endergonic at 523 K, suggesting that those models are less representative of partially oxidized Ag surfaces under some EO reaction conditions.^19,21^

**Table 1:** Bond Distance in O_2_* Species and Corresponding Frequencies for the Two Surfaces Shown in Figure 4a

species	O–O bond (Å)	O–O frequency (cm^–1^)
O_2_^–^	1.30	1155
O_2_^2–^	1.39	931
η^2^-O_2_–C_2_H_4_* on superoxo surface	1.46	785
η^2^-O_2_–C_2_H_4_* on peroxo surface	1.51	713

Figure 4b shows the free energy diagram for reactions between C_2_H_4_ and O* and O_2_* on the peroxo-containing surface (80 kPa O_2_, 0.75 kPa C_2_H_4_, 0.15 kPa EO, 0.0375 kPa AA, 523 K). The sequence begins with adsorption and activation of diatomic oxygen through either C_2_H_4_*-assisted dissociation or direct dissociation. Within the C_2_H_4_*-assisted dissociation pathway (blue traces), adsorbed peroxo (O_2_^2–^) reacts with C_2_H_4_* to form an η^2^-O_2_–C_2_H_4_ intermediate that reacts further and cleaves the O–O bond to yield O* and an oxometallacycle (OMC*, η^2^-O–C_2_H_4_), similar to the structure described by Linic and Barteau.^8,9^ For the direct dissociation pathway (red traces), C_2_H_4_* reacts with O* to form η^2^-O–C_2_H_4_ with a maximum free energy barrier indistinguishable from the C_2_H_4_*-assisted dissociation pathway within the accuracy of DFT (Figure 4b, C_2_H_4_*-assisted dissociation shows a barrier 5 kJ mol^–1^ greater than the direct dissociation pathway), which suggests that reactions of C_2_H_4_ with O* and O_2_* compete. Direct reaction between C_2_H_4_ and O_2_* carries the highest degree of rate control in the microkinetic model developed by Rangarajan and co-workers.^29^ Similarly, our results suggest that this step remains kinetically relevant but occurs in parallel with the C_2_H_4_ reaction with O*, and the contributions of these pathways depend on the reaction conditions (*vide infra*). Importantly, the barriers for the desorption of molecular oxygen far exceed those for the consumption of O_2_* by reactions with C_2_H_4_ through either pathway, consistent with the lack of detectable O_2_ desorption during transient cutoff experiments (Figures 3 and S9). The η^2^-O–C_2_H_4_ forms (bound adjacent to a liberated O*) and then reacts to yield either EO* or AA* by steps with relatively low free energy barriers (Δ*G*^‡^ is 28 kJ mol^–1^ for EO and 1 kJ mol^–1^ for AA, Figure 4b and Table S11). Finally, the O* liberated from the η^2^-O_2_–C_2_H_4_ can either react with a second equivalent of C_2_H_4_ to form an η^2^-O–C_2_H_4_ (Δ*G*^‡^ = 99 kJ mol^–1^) or reform O_2_* through recombination with another O* (Δ*G*^‡^ = 48 kJ mol^–1^). Notably, formation of η^2^-O–C_2_H_4_ on this surface proceeded with a significantly higher barrier (Δ*G*^‡^ = 94 kJ mol^–1^ higher) compared to the prior surface that had a higher total number of O*. This η^2^-O–C_2_H_4_ complex reacts through steps with greater barriers and preferentially forms AA (Δ*G*^‡^ = 48 kJ mol^–1^) by a pathway more favorable than that to produce EO (Δ*G*^‡^ = 95 kJ mol^–1^).

In comparison, the surface containing superoxo (O_2_^–^) species provides greater barriers for the analogous pathways to form EO and AA with a maximum Δ*G* (difference between the O_2_* surface and highest free energy intermediate/transition state) that is 25 kJ mol^–1^ greater than the comparable processes on the peroxo-containing surface (Figure S16 and Table S11). The lower reactivity of superoxo species relative to peroxo is consistent with the persistence of the superoxo band in the transient experiments shown in Figure 3.

As mentioned earlier, our calculations show differences in EO selectivity with a changing number of surface oxygen atoms. Catalytic cycles on both peroxo and superoxo surfaces have one EO generated from O_2_* and then a second from O*; therefore, the second EO comes from a less oxidized state of the surface. We estimated the relative preference for EO or AA formation by calculating the difference between the free energy barriers for EO and AA (ΔΔ*G*^‡^_EO-AA_). A large positive value of ΔΔ*G*^‡^_EO-AA_ corresponds to a higher EO formation barrier and, consequently, unfavorable EO formation. For both peroxo- and superoxo-containing surfaces, the ΔΔ*G*^‡^_EO-AA_ was higher on the surface with a lower total number of surface O* (ΔΔ*G*^‡^_EO-AA_ = 47 kJ mol^–1^ for peroxo and ΔΔ*G*^‡^_EO-AA_ = 29 kJ mol^–1^ on superoxo-containing surface) compared to the beginning of the reaction energy diagrams with a higher total number of surface O* (ΔΔ*G*^‡^_EO-AA_ = 27 kJ mol^–1^ for peroxo and ΔΔ*G*^‡^_EO-AA_ = 18 kJ mol^–1^ on superoxo-containing surface). The free energy to recombine 2O* to form O_2_* also becomes prohibitive after the formation of the first EO or AA product (Table S12, not shown in Figure 4b), preventing the formation of a second η^2^-O_2_–C_2_H_4_ intermediate. These calculations suggest that EO formation becomes more favorable with an increase in the total number of surface oxygen, which is generally consistent with the increase in selectivity with higher O_2_ pressures reported in Figure 1b and the decrease in EO selectivity as oxygen species are consumed, shown in Figure 3.

We also considered Eley–Rideal (ER) pathways involving gas C_2_H_4_ reacting with O*^13,15^ and O_2_* over peroxo- and superoxo-containing surfaces; however, these processes exhibit less favorable reaction free energies compared to Langmuir–Hinshelwood (LH) pathways (Section S4.1 and Figure S15 and Tables S7 and S8). Consequently, the LH pathways likely dominate. The conclusions agree with findings from Linic et al. obtained for reconstructed Ag surfaces with lower O* coverages.^42^ Yet, Figures 3 and 4 give strong evidence that surfaces with higher subsurface O content (e.g., Ag_2_O models used here that contain higher total oxygen content than those by Linic) give lower activation free energies for C_2_H_4_*-assisted activation of O_2_* and direct dissociation of O_2_*, which we interpret as a consequence of the greater extent of charge transfer facilitated by subsurface oxygen atoms.^41^

These calculations suggest that on oxidized Ag surfaces, direct reactions between C_2_H_4_* and O_2_^2–^ occur in tandem with reactions between C_2_H_4_* and O* to provide pathways to activate and consume O_2_ and create EO. In addition, direct dissociation of O_2_* proceeds endergonically and reversibly (Δ*G*^‡^ = 48 kJ mol^–1^ for peroxo and Δ*G*^‡^ = 38 kJ mol^–1^ for superoxo), which agrees with the detection of O_2_* species (840 cm^–1^) that form from O* (630 cm^–1^) by *in situ* Raman spectroscopy after heating O*-covered silver to 523 K in pure helium (Section S2.4 and Figure S9; note that these Raman peaks shift slightly compared to the peaks in Figure 1a due to differences in coverages of oxygen- and ethylene-derived intermediates). These results strongly suggest that the O* reforms O_2_* at rates comparable to those of other surface reactions on oxidized Ag surfaces, which contradicts the long-standing assumption that the O_2_* dissociates irreversibly during epoxidation of ethylene. Furthermore, the comparisons among reaction coordinates for distinct stoichiometries of oxidized Ag surfaces suggest that surfaces with a greater oxygen content bind O_2_* and C_2_H_4_-derived intermediates more favorably and can lead to greater rates of EO formation, which agrees with transient *operando* Raman experiments (Figures 2 and 3).

**Figure 5:**
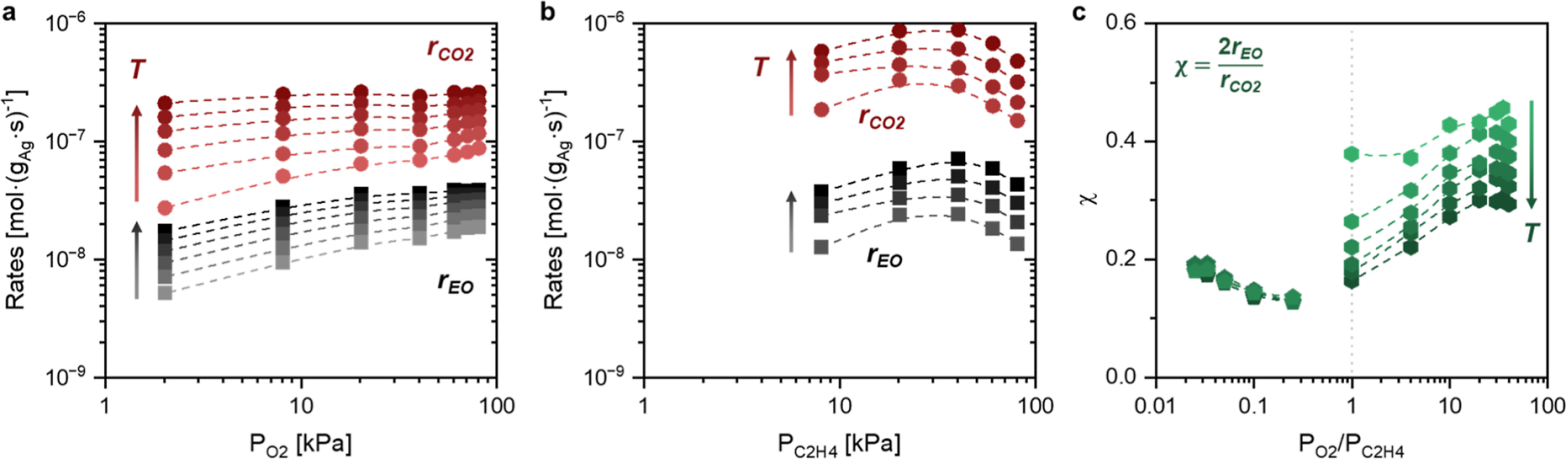
Steady-state formation rates of EO (■) and CO_2_ (●) as functions of (a) O_2_ pressure (2 kPa C_2_H_4_, 473–523 K in 10 K increments) and (b) C_2_H_4_ pressure (2 kPa O_2_, 508–523 K in 7 K increments) across a range of reactor temperatures. (c) Values of χ [χ = 2·*r*_EO_/*r*_CO_2__, calculated from rates reported in (a,b)] as functions of the ratio of O_2_ to C_2_H_4_ pressures (*P*_O_2__/*P*_C_2_H_4__). Catalyst: 35 wt % Ag/SiC.

**Figure 6:**
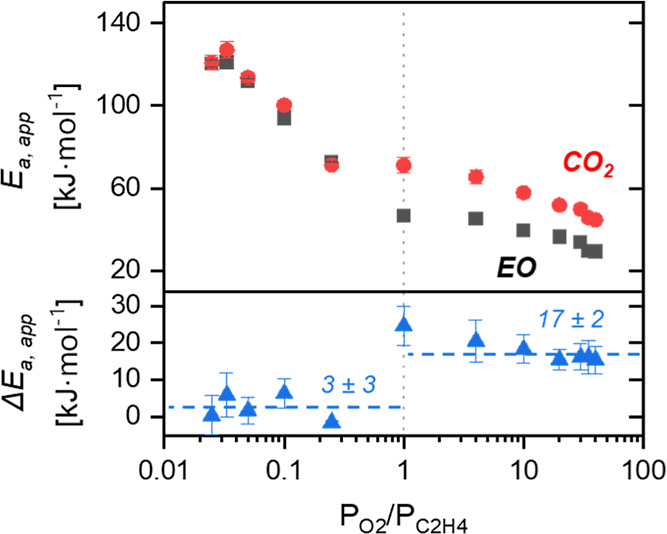
Apparent activation energies (*E*_a,app_) for the formation of EO (■) and CO_2_ (●) together with the corresponding differences (Δ*E*_a,app_, ▲) as functions of the ratio of O_2_ to C_2_H_4_ pressures (*P*_O_2__/*P*_C_2_H_4__). Values calculated from steady-state rates measured across a range of different temperatures. Catalyst: 35 wt % Ag/SiC.

We performed steady-state rate measurements in a hydrodynamically ideal plug-flow reactor (PFR) to obtain reliable apparent activation energies (*E*_a,app_) for EO and CO_2_ formations across a wide range of conditions. Figure 5a,b shows that formation rates of EO and CO_2_ depend weakly on the variations in *P*_O_2__ and *P*_C_2_H_4__, which confirms our observations during *operando* Raman experiments (Section S2.1 and Figure S5) and prior studies for silver catalysts in the absence of chlorine promoters or at the lowest coverages of chlorine obtained.^3,43^Figure 5c shows that values of χ in O_2_-rich conditions increase with *P*_O_2__/*P*_C_2_H_4__ presumably due to increases in O_2_^2–^ coverages observed by Raman (Figure 1) and depend strongly upon temperature, which signifies the differences between *E*_a,app_ for EO and CO_2_ formation at these conditions. In contrast, values of χ in C_2_H_4_-rich conditions change modestly with *P*_O_2__/*P*_C_2_H_4__ and appear to be independent of temperature. These results suggest that bimolecular reactions between O_2_* and O* with C_2_H_4_* remain kinetically relevant across all conditions examined, and elementary steps with distinct barriers govern selectivity to EO on more (O_2_-rich conditions) or less (C_2_H_4_-rich conditions) oxidized surfaces. Figure 6 shows calculated *E*_a,app_ values for the formation of EO and CO_2_ and reveals that *E*_a,app_ for both decrease systematically with increasing *P*_O_2__/*P*_C_2_H_4__. These trends suggest that the kinetically relevant processes possess lower barriers on surfaces with higher O_2_^2–^ coverages. Furthermore, *E*_a,app_ values are similar for both pathways (i.e., Δ*E*_a,app_ = 3 ± 3 kJ mol^–1^) on the most reduced surfaces (C_2_H_4_-rich conditions), whereas the more oxidized Ag surfaces present a notably lower *E*_a,app_ for EO formation that introduces a difference between the barriers (Δ*E*_a,app_ = 17 ± 2 kJ mol^–1^). These trends both in values of *E*_a,app_ and Δ*E*_a,app_ appear consistent with the similar intrinsic energy barriers (difference of <20 kJ mol^–1^) for EO and AA predicted from the DFT-derived reaction coordinates (Figures S17 and S18 and Section S4.2 and Table S10), although the computed barriers for AA formation (subsequently forming CO_2_) are generally lower. The selectivity differences between model predictions and experimental observations plausibly reflect differences between the surface structure under reaction conditions (likely disordered Ag_2_O_≤1_) and the Ag_2_O model surfaces used here to assess the effects of subsurface oxygen on C_2_H_4_ reactions with O* and O_2_*. Nevertheless, both computational and experimental results suggest that the O_2_-rich conditions result in the formation of surface peroxides that contribute to the formation of EO, and the coverage of peroxides should increase with increasing oxygen pressure (Figure 4a).

We next derived analytical rate expressions for the formation of EO and AA (Section S5), based on the minimum energy pathways depicted in the reaction coordinates (Figure 4b and Section S4.2 and Table S11). Comparisons of these expressions to steady-state rates and χ values demonstrate that apparent rate constants must depend strongly on oxygen coverages, which agrees with the computed reaction coordinates and measured trends in *E*_a,app_ values. Specifically, these expressions predict that χ will asymptotically approach constant values when EO and CO_2_ form primarily from a subset of reactive oxygen species (e.g., O* at low *P*_O_2__/*P*_C_2_H_4__ or O_2_^2–^ at high *P*_O_2__/*P*_C_2_H_4__), and there will be a transition region involving contributions from both O* and O_2_^2–^ where χ gradually increases as *P*_O_2__/*P*_C_2_H_4__ and O_2_^2–^ coverages increase. This is consistent with the DFT-predicted increased preference for EO formation over surfaces containing a higher total of O-containing species (Figure 4b). Furthermore, inspection of these rate expressions (eqs S5.20–S5.22) and calculated reaction energy coordinates (Figures S17 and S18) indicate that *E*_a,app_ values will decrease as O_2_^2–^ coverages increase as a consequence of greater ratios of *P*_O_2__ to *P*_C_2_H_4__. Significantly, the agreement between computed reaction coordinates for distinct stoichiometries of oxidized Ag surfaces and measured trends in *E*_a,app_ suggest that surfaces with greater oxygen content bind O_2_* and C_2_H_4_-derived intermediates more favorably and give greater rates of EO formation, as shown also in transient *operando* Raman experiments (Figures 2 and 3).

The observations from *operando* Raman spectroscopy, *ab initio* calculations on model surfaces, and steady-state kinetic measurements give strong evidence that the catalytic cycle initiates by either assisted or direct O_2_* activation to form O* and η^2^-O–C_2_H_4_, which further decomposes to EO and AA on the peroxo-containing surface (Figures 3,S4, and 4b). Reactions between C_2_H_4_ and either O_2_^2–^ or O* on silver surfaces with high-coverages of subsurface and surface oxygen offer pathways with the greatest energy preference to form EO.

## 3 Conclusions

Here, we combined DFT, kinetics, and spectroscopic measurements to investigate the mechanism of EO formation over unsupported Ag catalysts. Through *operando* Raman spectroscopy, steady-state experiments, and DFT-calculated reaction coordinates on model surfaces, we observed that an increase in EO selectivity was correlated with an increase in oxygen coverage. Higher O_2_ pressures and oxygen coverages led to an increase in O_2_* species detected by *operando* Raman spectroscopy that correlated with EO selectivity. Transient kinetic experiments where O_2_ was cut off from the reactant gas mixture showed a concomitant decrease in surface peroxo species and EO selectivity, implicating reactions between ethylene and peroxo intermediates as a viable pathway for EO formation. Quantification of oxygen consumed in the transient experiments suggests that the Ag surface and subsurface contain substantial quantities of oxygen, motivating the use of an oxide slab model (Ag_2_O) to model reactions between ethylene and surface oxygen species. DFT calculations showed that the formation of surface superoxo and peroxo species is thermodynamically favorable on Ag_2_O slab models at relevant conditions. Comparisons among reaction coordinates showed that EO formation is more favorable over surfaces containing peroxo species, and in general, surfaces containing more oxygen led to higher EO selectivities.

The knowledge gained from this investigation of a relatively simple unsupported (and unpromoted) silver catalyst provides insight into the possible modes of action of promoters (e.g., chlorine, rhenium, etc.) during industrial processes. Our findings clearly demonstrate that selectivity to EO increases with the coverage of O_2_^2–^ on oxidized Ag surfaces, which strongly suggests that common promoters may provide benefits by facilitating the oxidation of Ag such that numbers of surface O_2_^2–^ increase and oxygen vacancies decrease. In the absence of promoters, the Ag surfaces clearly bind a few of these crucial O_2_^2–^ species and give lower selectivities to EO under the C_2_H_4_-rich conditions selected for ethylene epoxidation. Notably, the forms of Ag catalysts optimized to give high EO selectivities within C_2_H_4_-rich conditions used in industry contain multiple promoters,^1^ including significant Cl content, which oxidizes the Ag surface. The approach used here to relate coverages of reactive forms of oxygen to rates and selectivities will remain crucial to validating these hypotheses. More broadly, we anticipate that these insights and the methodology developed here will expand the knowledge of partial oxidation reactions of hydrocarbons and elucidate strategies to promote metal catalysts for these chemistries.

## 4 Experimental Section

### 4.1 Materials

Polycrystalline Ag nanoparticles with diameters in the range of 80–100 nm (US Research Nanomaterials, 99.99%) were used throughout this work. The reported synthesis method of these particles forms a layer of organic ligand upon the surface of the nanoparticles,^44^ which is removed by *in situ* thermal treatments. These Ag nanoparticles were pelletized on their own or copelletized with silicon carbide nanoparticles (SiC, Beta, US Research Nanomaterials, 99%, ∼80 nm, cubic) and sieved to desired sizes before being loaded into reactors. Details of the preparation of Ag catalysts for different reactors are described in the corresponding experimental sections (*vide infra*).

Helium (He, Airgas, UHP grade), oxygen (O_2_, Airgas, UHP grade), ethylene (C_2_H_4_, Airgas, UHP grade and 10% in balanced He, certified standard grade), and hydrogen (H_2_, Airgas, UHP grade) were used to create mixtures for ethylene partial oxidation reactions and oxidative and reductive treatment of silver catalysts. O_2_ was fed through a hydrocarbon-moisture trap, C_2_H_4_ was fed through an oxygen-moisture trap, and He and H_2_ were fed through oxygen-hydrocarbon-moisture traps for further purifications. EO (Airgas, 0.5% in balanced He, certified standard grade) and CO_2_ (Airgas, 1.0% in balanced He, certified standard grade) were used to create calibration curves for product analysis in gas chromatography (GC).

### 4.2 Operando Raman Spectroscopy

#### 4.2.1 Instrumentation and Data Acquisition

The *operando* Raman spectroscopy integrates a Raman spectrometer with an *in situ* reaction cell and an online GC machine to simultaneously collect information on catalytic surface and reaction products. Raman spectroscopy was performed using a Raman spectrometer (Renishaw, InVia) equipped with a charged-couple detector and using a 532 nm laser adjusted to deliver low power densities at the sample (0.025–0.25 mW/m^2^). The laser power at the sample stage was measured directly with a power meter (Gentec-EO, PRONTO-Sl). All spectra were obtained with a long 50× objective and using a cell designed for *in situ* measurements within reactive environments (Linkam CCR1000 cell, ∼5 cm^3^ volume) equipped with a quartz window.

Reported Raman spectra reflect coadded measurements acquired for 30 s (0.2 s per measurement and then coadded 150 measurements to form 1 spectrum). Due to a short buffer time between the acquisition of each spectrum, the time resolution of Raman spectra is ∼45 s. Spectra were collected with a resolution of ∼1.5 cm^–1^, and the accuracy of the reported features is judged to be similar based on calibrations performed using the 520 cm^–1^ feature of a Si(111) wafer. In addition, spectra were obtained from the top surface of a packed catalyst bed; therefore, the composition of the gas reflects the feed stream, and consequently, the readsorption of products (e.g., EO, CO_2_, and water) was considered negligible. Raw data of the time-resolved Raman spectra were first processed using the WiRE 5.4 software (Renishaw) for baseline subtraction and peak fitting. The peak areas obtained through fitting were further processed in *Origin* (OriginLab) to perform summation, normalization (based on the area of peak in the range of 100–200 cm^–1^), averaging, and calculation of standard deviations. Detail procedures and parameters used for data processing are shown in Section S1.1 and Table S1.

Gases were delivered to the *in situ* cell by mass flow controllers (MFC, Alicat MC-50 sccm) through a gas manifold. The composition of gases leaving the *in situ* cell was analyzed using an online GC machine (990 Micro GC, Agilent) equipped with 3 parallel channels of thermal conductivity detectors (TCDs) and He as the reference gas. Each channel utilizes a designated set of columns and GC parameters to optimize the separation of specific analytes. Channel A uses a molecular sieve 5A SS column (10 m × 0.25 mm × 30 μm) to separate O_2_ from other analytes; Channel B uses a PoraPLOT Q UM column (10 m × 0.25 mm × 8 μm) to separate C_2_H_4_ and CO_2_ from other analytes; and Channel C uses a CP-WAX 52 CB column (10 m × 0.25 mm × 1.2 μm) to separate EO from other analytes. Additionally, Channels A and B are equipped with precolumns (CP PoraBOND Q, 1 m × 0.25 mm × 3 μm) and back-flush functions to prevent water from entering and blocking the pores of the main columns. Details of GC parameters and the retention times (τ) for analytes are listed in Table S2.

#### 4.2.2 Preparation of Pure Silver Catalysts

Unsupported pure silver catalyst was used for Raman spectroscopy to avoid any interference from the supports. The Ag nanoparticles were formed into aggregates (pellets) by applying high pressures (∼25 MPa) for ∼2 min using a pellet press (Carver, Model C) to produce structures with a cross-sectional area of ∼6 mm^2^ and a thickness of ∼1 mm. Approximately 0.25 g of these aggregates were loaded into the crucible of the in situ cell. A sequential oxidative–reductive pretreatment was then performed with the intent to remove all carbonaceous surface residues and subsequently reduce the catalyst back to the metallic state. Oxidative pretreatment was performed under O_2_ flow (101 kPa O_2_, 673 K, 4 h), and reductive pretreatment was performed under H_2_ flow (101 kPa H_2_, 673 K, 16 h). The total flow rate for the pretreatments was fixed at 30 sccm, and a ramp rate of 5 K·min^–1^ was used for the heating and cooling processes between ambient temperature and 673 K.

#### 4.2.3 Steady-State Operando Experiments

Steady-state *operando* experiments were performed at 523 K and a total pressure of 101 kPa across a range of *P*_O_2__/*P*_C_2_H_4__ values from 0.025 to 40. The total flow rate of the gas stream was fixed at 50 sccm. Reactions under oxygen-rich conditions were performed at 2 kPa C_2_H_4_ with 2–80 kPa O_2_, while those under ethylene-rich conditions were performed at 2 kPa O_2_ with 2–80 kPa C_2_H_4_. We began with the highest *P*_O_2__/*P*_C_2_H_4__ (i.e., 40) and gradually shifted toward the lowest *P*_O_2__/*P*_C_2_H_4__ (i.e., 0.025). At each composition, spectra were acquired continuously for at least 24 h in duration until both the *in situ* Raman spectra and reaction rates no longer changed (Figure S2), which demonstrated that these measurements represent the kinetics on the steady-state structure of the operating Ag catalyst and at the corresponding coverages of surface intermediates.

#### 4.2.4 Transient Operando Experiments

Transient experiments were performed at 523 K and a total pressure of 101 kPa. Typically, Ag catalysts were first exposed to 80 kPa O_2_ and different levels of *P*_C_2_H_4__ (0.75, 1, or 2 kPa C_2_H_4_) until stable reaction rates and surface coverages were observed. Under these conditions, the Ag surface would bind high coverages of diatomic oxygen species. Then, the inlet streams were replaced by the respective dilute C_2_H_4_ flows (i.e., 0.75, 1, or 2 kPa C_2_H_4_) or pure helium (as control experiments) without O_2_ for 1 h, after which the reaction conditions were switched back to the original O_2_-rich streams (80 kPa O_2_ and 0.75, 1, or 2 kPa C_2_H_4_) for 3 h. The total flow rates were generally fixed at 50 sccm, but the total flow rate of experiments involving 0.75 kPa C_2_H_4_ (including the corresponding control experiments) was fixed at 40 sccm with an intent to increase the conversion for better product detection in GC. Each set of transient *operando* experiment was repeated 3 times to ensure data replicability. Reported spectrokinetic data represent an average of three identical measurements with error bars showing one standard deviation.

### 4.3 Kinetic Measurement in the PFR

#### 4.3.1 Instrumentation and Data Acquisition

We built a homemade PFR equipped with an online GC (Agilent 6890) to achieve long time frame (∼weeks) kinetic measurements for the partial oxidation of ethylene over Ag catalysts. The catalysts were loaded to a 3/8-in. O.D. stainless-steel tubular reactor (Swagelok), which was placed in an aluminum clamshell heated by two cartridge heaters (Nexthermal, 1/4 in. diameter, 10 in. length, 250 W, 120 V AC power, 12 in. swaged fiberglass leads rated to 250 °C), and the temperature was regulated by a Watlow EZ-ZONE temperature controller.

Flow rates of the gaseous reactants were controlled by MFCs (Parker Porter, model 601) using a controller bus (Parker Porter, model CM-400). The gases were delivered to the reactor through 1/4 in. of O.D. stainless steel tubing (Swagelok), while the stream leaving the reactor was delivered to the loop of GC gas-sampling valve through 1/8 in. of O.D. stainless-steel tubing (Swagelok). Heat tapes (Omega) were used to heat the stream before and after the reactor, where temperatures of the heat tapes were set to 100 °C, controlled by a Variac voltage regulator, to prevent condensation of water in the tubing.

The GC machine was equipped with two parallel channels of the injector, column, and detector. The front set includes a split–splitless inlet, an HP-PLOT Q capillary column (30 m × 320 μm × 20 μm), and a flame ionization detector (FID). The back set includes a purged packed inlet, a Hayesep D 80/100 UM packed column (2 m × 1/8 in. × 2 mm), and a TCD. Details of the GC parameters are listed in Table S3.

#### 4.3.2 Preparation of 35 wt % Ag/SiC Catalysts

Supported silver catalyst was used for the kinetic measurements on PFR. The Ag nanoparticles were mixed with silicon carbide nanoparticles to form a 35 wt % Ag/SiC mixture. This mixture was formed into aggregates (pellets) by applying high pressures (∼25 MPa) for ∼2 min using a pellet press and was subsequently crushed and sieved to 80–120 mesh size (125–177 μm). Approximately 0.1–1.0 g of these aggregates was loaded into the stainless-steel reactor of the PFR system. A sequential oxidative–reductive pretreatment was then performed with the intent to first remove all carbonaceous surface residues and subsequently reduce the catalyst back to the metallic state. Oxidative pretreatment was performed under O_2_ flow (101 kPa O_2_, 673 K, 4 h), and reductive pretreatment was performed under H_2_ flow (101 kPa H_2_, 673 K, 16 h). The total flow rate for the pretreatments was fixed at 90 sccm, and a ramp rate of 5 K·min^–1^ was used for the heating and cooling processes between ambient temperature and 673 K.

#### 4.3.3 Steady-State Kinetic Measurements on PFR

Steady-state kinetic measurements carried out in the PFR system were performed at a total pressure of 101 kPa across a range of *P*_O_2__/*P*_C_2_H_4__ values from 0.025 to 40, which is consistent with the conditions applied in the *operando* Raman system. Reactions under oxygen-rich conditions were performed at 2 kPa C_2_H_4_ with 2–80 kPa O_2_, while those underethylene-rich conditions were performed at 2 kPa O_2_ with 2–80 kPa C_2_H_4_. In addition, we studied the dependency of rates on temperatures in which the range of temperature was 473–523 K (10 K per increment) for *P*_O_2__/*P*_C_2_H_4__ between 1–40 and 488–523 K (7 K per increment) for *P*_O_2__/*P*_C_2_H_4__ between 0.025–0.25.

We began with the highest *P*_O_2__/*P*_C_2_H_4__ (i.e., 40) condition following the oxidative–reductive pretreatments, heating the reactor under the corresponding reactant flow from ambient to 523 K with a ramp rate of 5 K·min^–1^, and then held at 523 K until steady-state rates were observed. After that, the temperature of the reactor was reduced incrementally (e.g., 10 K per increment in this condition) to 473 K, held for 4 h at each temperature, and then raised directly to 523 K from 473 K with a ramp rate of 5 K·min^–1^ before the composition of the gas stream was switched to a lower *P*_O_2__/*P*_C_2_H_4__. The same procedure of the temperature dependency experiments was followed for each *P*_O_2__/*P*_C_2_H_4__ until we arrived at the lowest *P*_O_2__/*P*_C_2_H_4__ (i.e., 0.025). The total flow rate of the gas stream was adjusted correspondingly between 60 and 180 sccm to ensure that we are operating at differential reactor conditions across all temperatures and *P*_O_2__/*P*_C_2_H_4__ while still obtaining good signal-to-noise ratio in the GC chromatograms to determine the formation rates.

## 5 Computational Section

### 5.1 DFT Calculations

We performed spin-polarized DFT calculations using the Vienna Ab initio Simulation Package (VASP), version 5.4.4.^45^ All geometry optimizations were performed using the spin-polarized generalized gradient approximation functional of Perdew–Burke–Ernzerhof (GGA-PBE)^46^ for the exchange–correlation potential coupled with Becke–Johnson damping [D3(BJ)vdw] dispersion correction.^47^ We used the projector-augmented wave method^48,49^ of core valence interactions and a plane wave cutoff energy of 400 eV. All structures were optimized to at least 10^–7^ eV and 0.03 eV/Å for energy and force convergence criteria, respectively. Since GGA functionals overestimate the O_2_ bond energies,^50^ we used the H_2_O formation energy [H_2(g)_ + 1/2O_2(g)_ = H_2_O_(g)_] from DFT and compared it to experimentally reported formation energy to correct the O_2_ gas energy. The free energy calculations were done at *P*_O_2__ = 80 kPa, *P*_C_2_H_4__ = 0.75 kPa, *P*_EO_ = 0.15 kPa, and *P*_AA_ = 0.0375 kPa; these partial pressures correspond to experimental conditions (25% C_2_H_4_ conversion, 40% EO selectivity; acetaldehyde is assumed to spontaneously combust into CO_2_ and H_2_O). Structures of all reaction intermediates and transition states are included in the atomic_structures.zip file (Supporting Information).

### 5.2 Surface Model

For surface calculations, we chose Ag_2_O(001) as a representative model for the top 1–2 nm of Ag nanoparticles, which maintain a silver core but are enveloped in an oxide film with a high content of surface and subsurface oxygen.^7,19,31,51−54^ Notably, the presence of both surface and subsurface oxygens has been linked to higher EO selectivities.^55^ To model this high O-content surface, we used the asymmetric 2 × 2 Ag_2_O(001) slab with five layers, and the bottom layer fixed. We added at least 12 Å of vacuum to all surface calculations and used Monkhorst–Pack *k*-points with a 3 × 3 × 1 mesh, which we confirmed to be sufficient via the *k*-point convergence calculation for Ag_2_O. The original 2 × 2 Ag_2_O(001) surface, as used by van Santen and co-workers,^13,15^ terminates with 8 silver and 4 oxygen atoms, with the four oxygen atoms positioned slightly above the 8 silver atoms. These four oxygen atoms are treated as the 4 “O*” atoms in van Santen’s and our work (see Figure 4, structure “1111”). We consider the 4 O* sites to be the four adsorption sites that can host three types of species: O*, O_2_*, or an oxygen vacancy O_vac_. Previously,^19^ we enumerated all symmetrically unique combinations of the three species on this surface and computed their free energies using eq 5.2.1
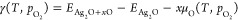
5.2.1where γ is the surface free energy,  is the total energy of the surface containing different O_2_*/O*/O_vac_ species,  is the total energy of the Ag_2_O(001) surface, and *x* is the number of added (or removed) oxygen atoms in the surface containing different O_2_*/O*/O_vac_ species. The range for *x* is from −4 (four vacancies) to +4 (four dioxygen). The term μ_O_ is the chemical potential of oxygen, which we relate to the pressure of O_2(g)_
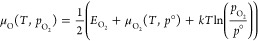
5.2.2
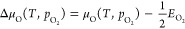
5.2.3where *E*_O_2__ is the energy of an isolated O_2_ molecule, and μ_O_2__ is calculated from NIST JANAF thermochemical tables^56^ at a standard state pressure (*p*°) of 101 kPa.

Free energies for all unique stoichiometries are plotted in Figure 4. Surfaces shown in the plot are denoted with four integers that represent different species adsorbed on the four adsorption sites: where “0” is O_vac_, “1” is O*, and “2” is O_2_*. For example, structure “0001” contains three O_vac_ and a single O*, while “1021” contains one O_vac_, two O*, and one O_2_*. Three surfaces appear on the convex energy hull, which are “0001”, “1021”, and “2111”. The surfaces we computed were only locally optimized, might not represent the global minima, and are treated as model surfaces to study the energetics of different O_2_* and O* species reacting with ethylene. We chose to compute reactions with ethylene on the two most thermodynamically stable surfaces that contain dioxygen, 1021 (referred to as the superoxo surface) and surface 2111 (referred to as the peroxo surface) because we experimentally observed that the two dioxygen species behaved differently when ethylene was fed in the reactor, where superoxo (experimental 990 cm^–1^) remain on the surface while peroxo (experimental ∼ 803 cm^–1^) are consumed (see Figure 3a). We assigned the computed surfaces as superoxo and peroxo based on the difference in the computed vibrational frequencies of O_2_* (1155 and 931 cm^–1^, respectively) reported in Table 1 as well as the charge difference of the O_2_* (see Tables S4 and S5). We computed the reaction pathways of superoxo and peroxo O_2_* reacting with ethylene to form EO (desired product) and AA (precursor in total combustion pathway) to understand how O_2_* speciation affects the desired and undesired pathways (Section S4).

### 5.3 Frequency Calculations

Harmonic vibrational frequency calculations for superoxo and peroxo surfaces and η^2^-O_2_–C_2_H_4_* intermediates on the two surfaces are reported in Table 1. Frequency calculations were performed with 0.015 Å displacements and an energy convergence criterion of 10^–7^ eV. Only atoms in the top layer (4 oxygen atoms and 8 silver atoms) were displaced. We confirmed that displacing an additional layer of atoms (a total of 8 oxygens and 16 silver atoms) did not change frequency values.

### 5.4 Reaction Barriers

Most of the reaction energy barriers were computed using the climbing image nudged elastic band (CI-NEB) method.^57,58^ For some of the elementary steps where we had issues converging CI-NEBs, we used the neb2dim.pl script (VTST Tools) on the previous NEB calculation to generate the inputs for a dimer calculation.^59^ We used convergence criteria of 10^–7^ eV and 0.03 eV/Å for energies and forces, respectively, for both methods, as implemented in the VTST Tools package.^58,59^ Transition state structures are provided in the atomic_structures.zip file (Supporting Information).

## Supporting Information Available

Additional experimental details for Raman spectroscopy, kinetic measurements in PFR, and Bader charge analysis; steady-state and transient kinetics collected by operando Raman spectroscopy; estimation of thickness of the oxidized silver surface from transient experiments; evidence for O* recombination; dependency of rates on flow rate, conversion, and temperature in PFR; measured apparent activation energies; simulated reaction pathways, energies, and barriers for ethylene epoxidation on different surfaces; and derivation of analytical rate expressions (PDF)
Structures of all reaction intermediates and transition states (ZIP)
