# Supplementary material for: Reactions of Surface Peroxides Contribute to Rates and Selectivities for C2H4 Epoxidation on Silver
Source: ACS Catal. 2025 Jan 9;15(2):1387–98. doi: 10.1021/acscatal.4c06945 (PMC11744664; doi:10.1021/acscatal.4c06945)
Supplement: Supplementary file 1 — cs4c06945_si_001.pdf [file cs4c06945_si_001.pdf]

Supporting Information

Reactions of Surface Peroxides Contribute to Rates and Selectivities for C_2H_4 Epoxidation on Silver

Ching-Tien Chen[1†&], Anna Sviripa[2†], Sugandha Verma[2], Christopher Paolucci[2*], David Flaherty[1*]

*Corresponding authors: cp9wx@virginia.edu (C.P.); dflaherty3@gatech.edu (D.W.F.)

[†]These authors contributed equally to this work.

[1]School of Chemical and Biomolecular Engineering, Georgia Institute of Technology; Atlanta, Georgia, 30332, United States

[2]Department of Chemical Engineering, University of Virginia; Charlottesville, Virginia, 22904, United States

[&]Present address: Department of Chemical Engineering, National Tsing Hua University, Hsinchu City, 300044, Taiwan

Section S1. Supplementary Methods

S1.1. Operando Raman Spectroscopy

Raw data of the time-resolved Raman spectra was first processed using a series of built-in functions in the WiRE 5.4 software (Renishaw) to subtract the baseline and create user-defined peaks to fit each spectrum. For baseline subtraction, we utilized the *subtract baseline* function and chose the intelligent fitting mode, where the intelligent polynomial with a polynomial order of 11 was selected as the type of baseline for all spectra in this study, and the noise tolerance was set to 1.50. We then utilized the *curve fit* function with designated parameters to create 22 different user-defined peaks for optimal fitting of the baseline-subtracted Raman spectra, as some of the Raman features are broad bands with shoulders that cannot be fitted using a single user-defined peak. We set a linear baseline for curve fitting with a floating slope of -0.02 ~ +0.03 and a floating offset between -200 ~ -40. The combination of Gaussian and Lorentzian peaks was enabled in the curve fitting parameters to increase flexibility in peak shapes. Peak heights were set to be floating between 0 to 3,000,000. Additionally, the center and width of the user-defined peaks are listed in **Table S1**. When running the curve fit function, we set a value of 0.01 for the tolerance level, a maximum of 200 iterations for fitting each spectrum, using an even weighting model.

Table S1. Curve fitting parameters for baseline-subtracted Raman spectra

Defined peaks	Peak center [cm^{-1}]		Peak width [cm^{-1}]		Raman band [cm^{-1}]
	Lower limit	Upper limit	Lower limit	Upper limit	
1	100	130	5	30	100 ~ 200 (artificial)
2	130	170	5	50	
3	210	260	5	40	200 ~ 700 (O*)
4	330	380	5	70	
5	440	480	5	40	
6	510	550	5	60	
7	600	700	5	80	
8	630	660	5	50	
9	690	720	5	50	700 ~ 1200 (O_2*)
10	730	770	5	100	
11	790	820	5	70	
12	820	850	5	70	
13	850	870	5	60	
14	875	900	5	60	
15	890	970	5	70	
16	980	1000	5	50	
17	1010	1065	5	80	
18	1080	1120	5	100	
19	1160	1220	5	50	
20	1300	1350	5	120	1200 ~ 1800 (C_2H_4*)
21	1420	1460	5	130	
22	1550	1650	5	130	

We note that there is a weak Raman feature at ~1603 cm^{-1} that appears when the Raman feature at ~803 cm^{-1} is strong (Main text, Fig 1a). We considered this feature as an overtone of 803 cm^{-1} and is not related to C_2H_4-derived surface intermediates, as this feature also presents when unsupported Ag nanoparticles were oxidatively treated at 101 kPa and 523 K without any C_2H_4 in the stream (**Fig. S1**).

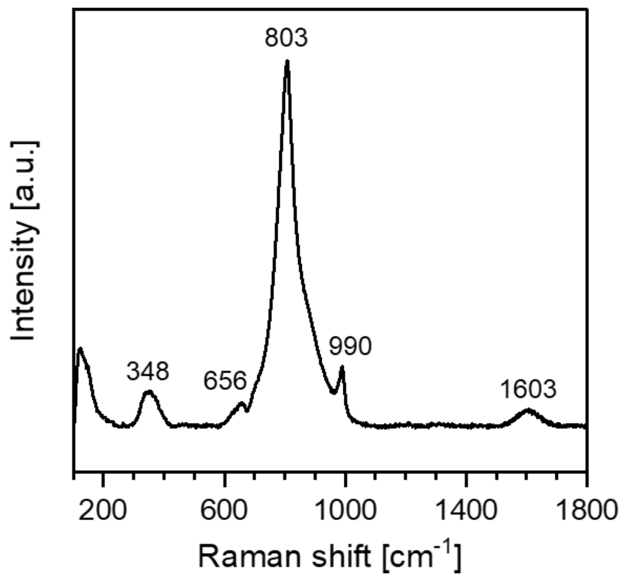

Fig. S1. *In situ* Raman spectrum of the unsupported Ag nanoparticles exposed to a 101 kPa O_2 flow at 523 K for 3 h. Flowrate of the O_2 stream is set to 30 sccm.

After collecting the peak area and intensity of each user-defined peak as a function of time, these data were exported as .txt files and then further processed in *Origin* (Originlab) to perform summation, normalization, averaging, and calculation of standard deviations. Reported normalized peak areas for O*, O_2*, and C_2H_4* represent the sum of peak areas of those user-defined peaks in ranges of 200 ~ 700, 700 ~ 1200, and 1200 ~ 1800 cm^{-1}, respectively, normalized by the area of peaks in the range of 100 ~ 200 cm^{-1}, which is an artificial feature that appears in every sample (not just Ag catalysts) since the wavenumber is approaching the lower limit of the scanning range of our Raman spectrometer. The density of data points (e.g., peak area and intensity of Raman features) was reduced by 4-folds by averaging every 4 consecutive data points; as a result, the level of noise was reduced, and the time resolution of Raman spectra changed from ~45 seconds to ~180 seconds, which became consistent with the time resolution of GC data.

Table S2. GC parameters for *operando* Raman spectroscopy

Channel	A	B	C
Column	MS5A SS (10×0.25×30)	PoraPLOT Q UM (10×0.25×8)	CP-WAX 52 CB (10×0.25×1.2)
Pre-column (for backflush)	CP PoraBOND Q (1×0.25×3)	CP PoraBOND Q (1×0.25×3)	None
Sample inlet temperature	30ºC	30ºC	30ºC
Sampling time	30 s	30 s	30 s
Injector temperature	110ºC	110ºC	110ºC
Injection time	40 ms	40 ms	40 ms
Backflush time	8 s	8 s	N/A
Column temperature	80ºC	60ºC	60ºC
Column pressure (static mode)	29 psi	21.8 psi	21.8 psi
Run time	140 s	50 s	35 s
τ (O_2)	0.355 ~ 0.370 min	N/A	N/A
τ (C_2H_4)	N/A	0.470 ~ 0.480 min	N/A
τ (CO_2)	N/A	0.384 ~ 0.394 min	N/A
τ (EO)	N/A	N/A	0.480 ~ 0.505 min

N/A: not available; Although TCD was used to detect the compounds in the gas stream, some compounds did not appear in the chromatogram because they were backflushed, while some compounds were unable to be separated from other analytes due to column properties.

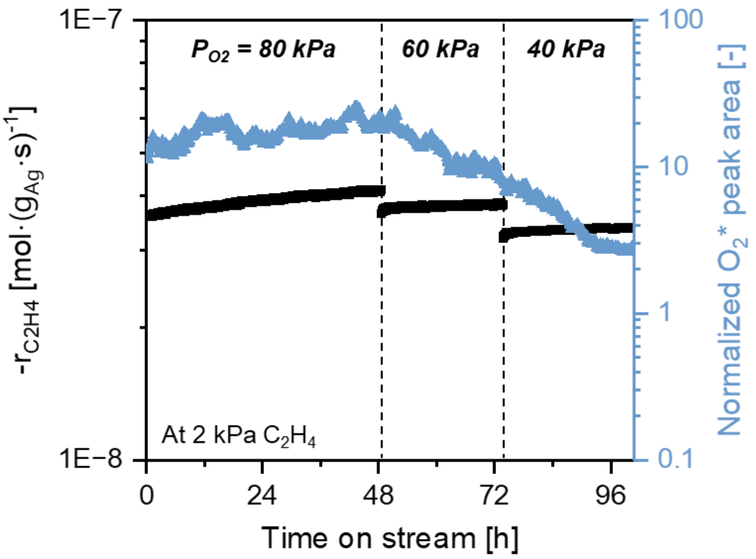

Fig. S2. Time-resolved *operando* Raman spectroscopy to determine the time for approaching steady-state operations. Instantaneous changes in ethylene reaction rates (black) and Raman peak area of surface diatomic oxygens (light blue) as a function of time when partial pressures of O_2 were changed at 2 kPa C_2H_4 and 523 K.

S1.2. Kinetic measurements in PFR

The information of GC operation parameters, columns, and retention times of analytes used in the PFR system is shown in **Table S3**.

Table S3. GC parameters for kinetic measurements in PFR

Channel	Front	Back
Gas sampling loop volume	1 mL	
Sample load time	0.5 s	
Inject time	0.1 s	
Inlet type	Split-splitless	Purged packed
Inlet temperature	220ºC	220ºC
Split ratio	30 (split mode)	N/A
Column	capillary HP-PLOT Q (30×320×20)	Packed Hayesep D (2×1/8×2)
Column flow	2.6 $mL \cdot min^{-1}$	20 $mL \cdot min^{-1}$
Oven temperature program	Initial: 35ºC, hold 0 min<br>Ramp 1: heat to 65ºC (30ºC$\cdot min^{-1}$), hold 3 min<br>Ramp 2: heat to 180ºC (25ºC$\cdot min^{-1}$), hold 1 min<br>End (cool to 35ºC before the next injection)	
Detector	FID	TCD
Carrier gas/Reference gas	Ar	H_2
Detector temperature	245ºC	250ºC
(FID) H_2 flow	40 $mL \cdot min^{-1}$	N/A
(FID) Air flow	450 $mL \cdot min^{-1}$	N/A
(TCD) reference flow	N/A	50 $mL \cdot min^{-1}$
Makeup flow	50 $mL \cdot min^{-1}$ (Ar)	2 $mL \cdot min^{-1}$ (H_2)
τ (O_2)	N/A	0.52 ~ 0.63 min
τ (C_2H_4)	1.49 ~ 1.80	N/A
τ (CO_2)	N/A	1.40 ~ 1.60 min
τ (EO)	6.70 ~ 6.80	N/A
τ (H_2O)	N/A	4.40 ~ 4.90 min

N/A: not available; We are unable to determine the retention time of C_2H_4 and EO in the TCD chromatograms because H_2 was used as the carrier gas and reference gas, which reacts with C_2H_4 and EO to produce multiple compounds.

S1.3. Bader charge analysis

We performed Bader charge analysis using the method developed by Henkelman et.al.[1-2] Bader charge analysis of an isolated O_2(g) molecule showed charges of 6.1 and 5.9 e^- on the two O atoms. We use these values as a benchmark for comparison of charges for O_2* (superoxo, peroxo) species before and after the reaction with C_2H_4 to form η^2-O_2-C_2H_4* (see **Fig. S3** and **Tables S4** and **S5**). We tested the charge sensitivity to the grid size used in the Bader charge analysis and found that doubling it did not affect the charge assignment.

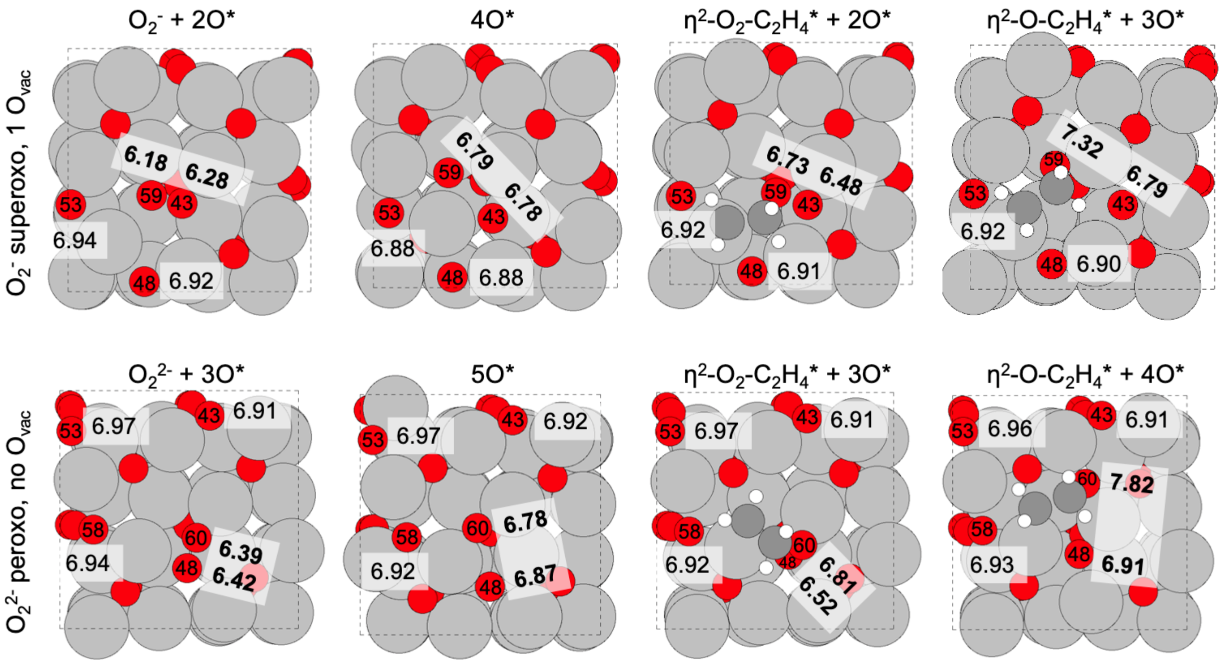

Fig. S3. Bader charge analysis (shown in white translucent boxes next to corresponding oxygens) for all surface oxygen species. The top row is the surface with one vacancy and superoxo O_2^-, and the bottom row is the surface with no vacancies and peroxo O_2^{2-}. Bader charges for atoms in isolated O_2(g) were 6.1 and 5.8 e^-. Spheres represent silver (light gray), oxygen (red), carbon (dark gray), and hydrogen (white) atoms.

Table S4. Bader charge analysis for the four surface oxygens (one O_2^- and two O*) for the surface initially containing the superoxo. Indices of atoms that initially make up the O_2^- are highlighted in bold.

Atom index	O_2^-	2O*	η^2-O_2-C_2H_4*	η^2-O-C_2H_4*+O*	η^2-O-C_2H_4*
**59**	**6.18**	**6.79**	**6.73**	**7.32**	**-**
**43**	**6.28**	**6.78**	**6.48**	**6.79**	**7.12**
53	6.94	6.88	6.92	6.92	6.95
48	6.92	6.88	6.91	6.90	6.94

Table S5. Bader charge analysis for the four surface oxygens (one O_2^{2-} and two O*) for the surface initially containing the peroxo. Indices of atoms that initially make up the O_2^{2-} are highlighted in bold.

Atom index	O_2^{2-}	2O*	η^2-O_2-C_2H_4*	η^2-O-C_2H_4*+O*	η^2-O-C_2H_4*
**60**	**6.39**	**6.78**	**6.81**	**7.28**	**-**
**48**	**6.42**	**6.87**	**6.52**	**6.91**	**7.16**
58	6.94	6.92	6.92	6.93	6.96
53	6.97	6.97	6.97	6.96	6.93
43	6.91	6.92	6.91	6.91	6.90

Section S2. Supporting Information for *operando* Raman Spectroscopy

S2.1. Steady-state measurements

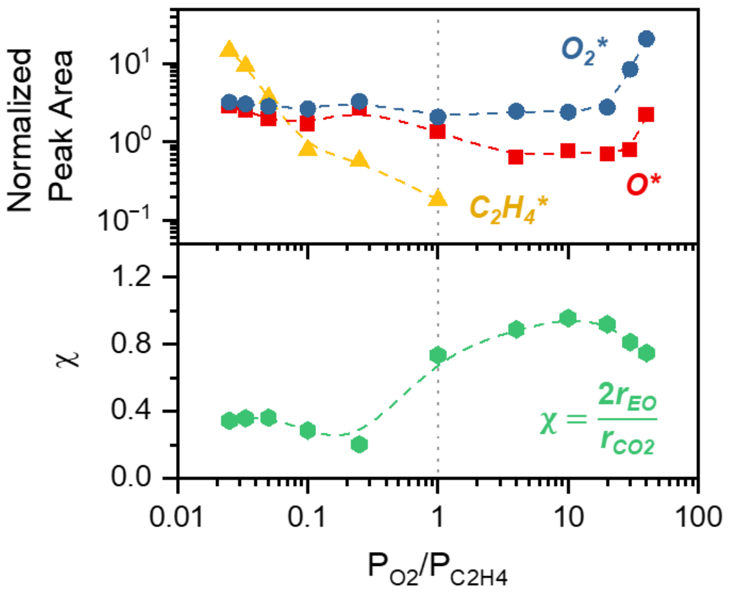

Fig. S4. Correlated changes in Raman features and ratios of the EO to CO_2 formation rates (defined as $\chi = 2 \cdot r_{EO}/r_{CO2}$) as functions of the ratios of O_2 to C_2H_4 partial pressures (P_{O2}/P_{C2H4}, 2 – 80 kPa O_2, 2 – 80 kPa C_2H_4).

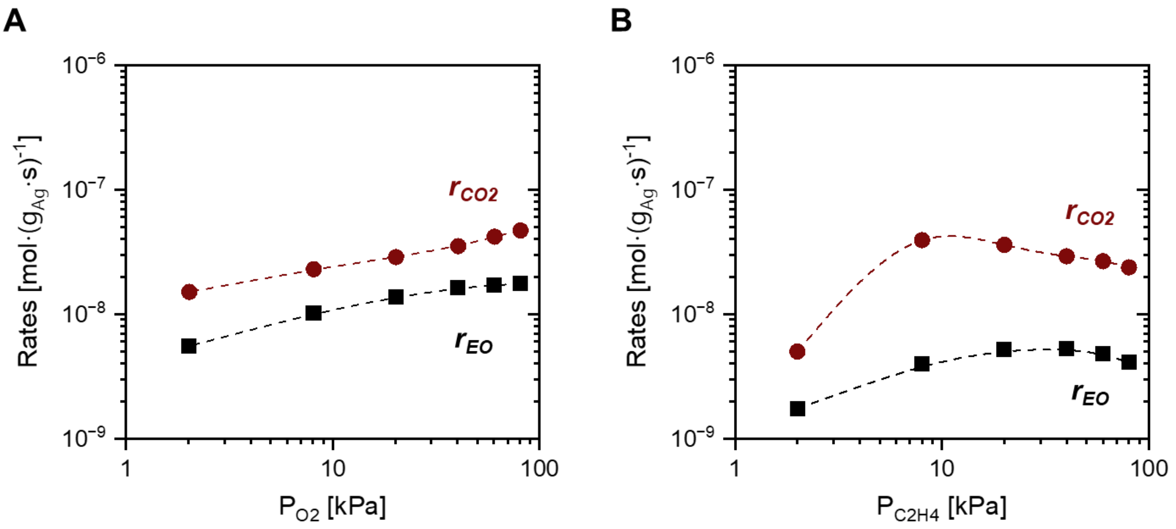

Fig. S5. Steady-state rates at 523 K obtained in *operando* Raman spectroscopy. Dependency of rates on (A) partial pressure of O_2 at 2 kPa C_2H_4, and (B) partial pressure of C_2H_4 at 2 kPa O_2.

S2.2. Transient measurements

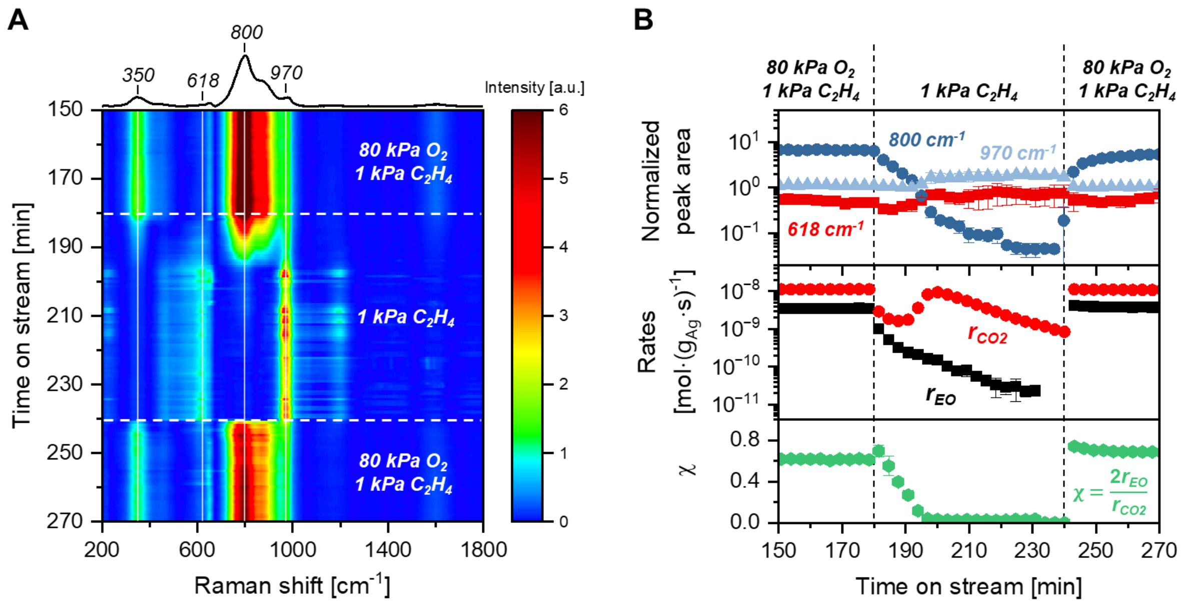

Fig. S6. Transient *operando* Raman spectroscopy obtained following the removal of O_2 from the reactant stream of a catalyst operating at steady-state (80 kPa O_2, 1 kPa C_2H_4, 523 K). (A) Heat map of time-resolved Raman spectra (spectrum at the top represents steady-state at 150 min), and (B) correlated changes in Raman features, formation rates of EO and CO_2, and values of χ as a function of time. Data points represent an average of three identical measurements with error bars showing one standard deviation.

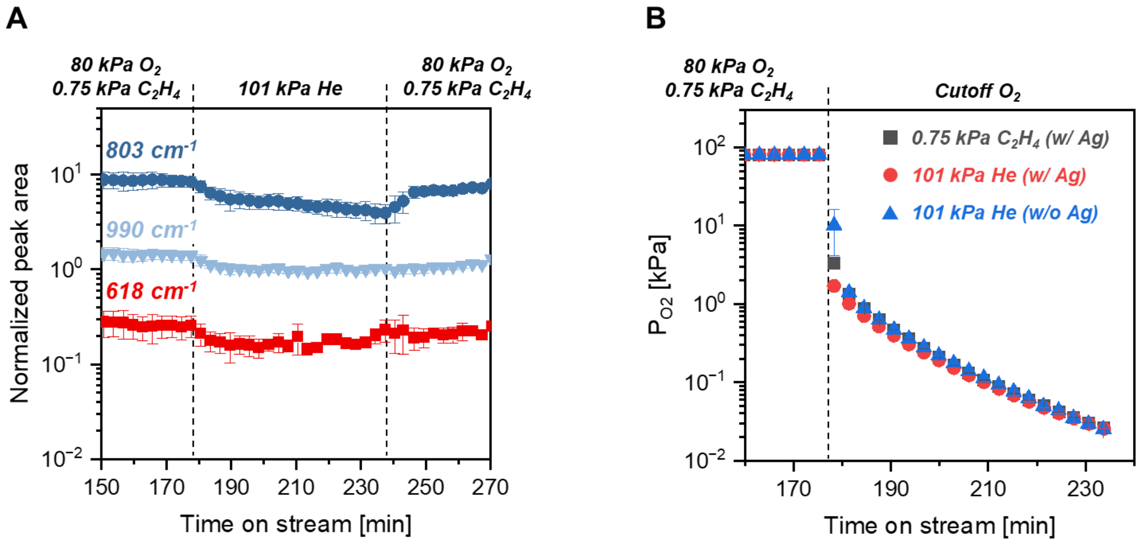

Fig. S7. Transient *operando* Raman spectroscopy obtained following the removal of O_2 from the reactant stream of a catalyst operating at steady-state (80 kPa O_2, 0.75 kPa C_2H_4, 523 K). (A) Changes in normalized peak area of Raman features when the reaction stream was replaced by pure He flow in the presence of Ag catalysts in the crucible of the *in situ* cell. (B) Decay of oxygen pressures when reaction streams were replaced by 0.75 kPa C_2H_4 or pure He flows with and without the presence of Ag catalysts in the crucible of the *in situ* cell.

S2.3. Thickness of the surface reconstructed layer on Ag nanoparticles

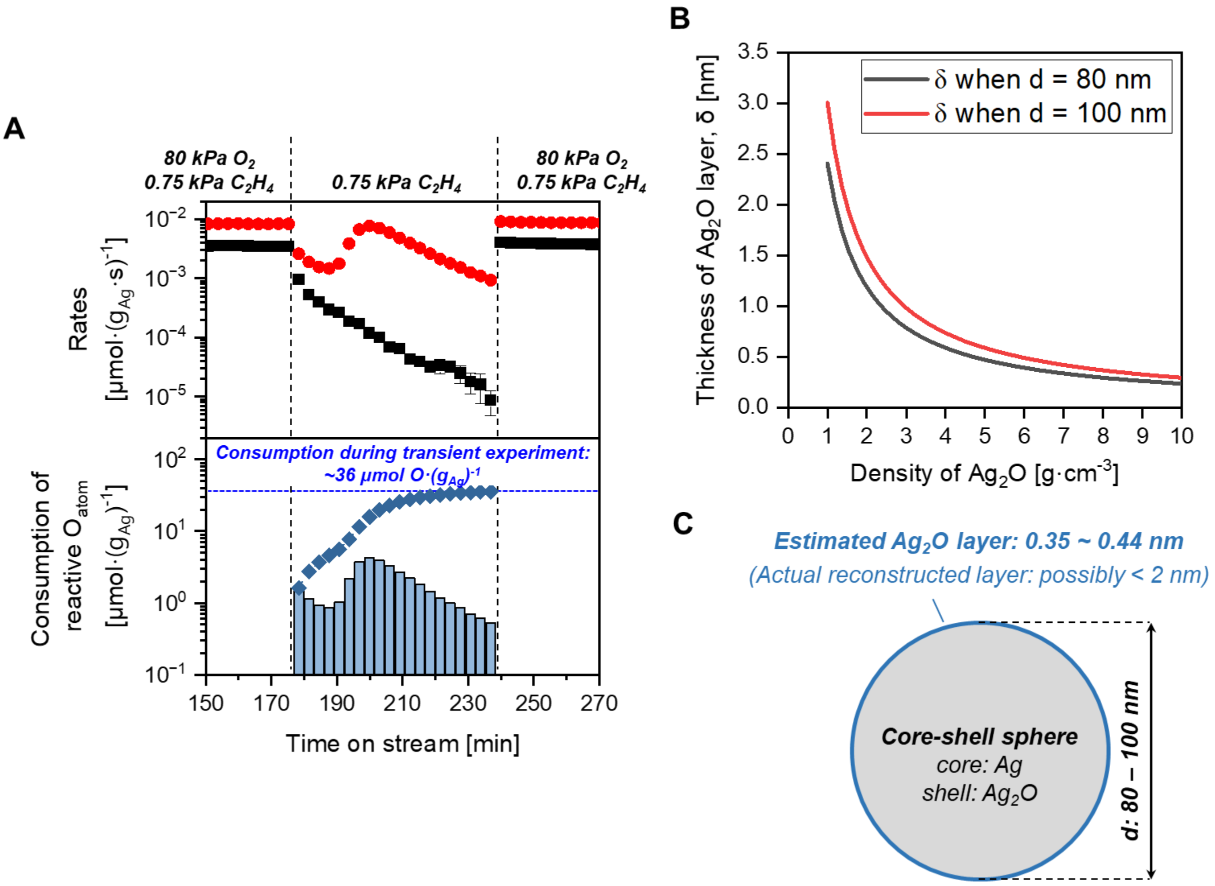

Fig. S8. Estimating the thickness of surface reconstructed layer on Ag from transient *operando* Raman experiments. (A) Number of reactive oxygen atoms on silver surface consumed during transient experiment (~60 min). (B) Change in δ as a function of Ag_2O density. (C) Schematic plot of the model used for estimation. Reaction flow was switched from an O_2-rich composition (80 kPa O_2 and 0.75 kPa C_2H_4) to a C_2H_4 flow (0.75 kPa C_2H_4) at 523 K. Reported data correspond to the average of 3 independent sets of experiment with identical conditions. Note that the estimated values for oxygen consumption should be a conservative lower bound of the reactive oxygen species because we did not perform the transient experiment until the C_2H_4 conversion achieves zero.

We used the following balanced chemical equations to estimate the consumption of surface reactive oxygen species during the transient period:

$$C_2H_4 + O \rightarrow EO \quad \text{(S2.3.1)}$$

$$0.5\ C_2H_4 + 3O \rightarrow CO_2 + H_2O \quad \text{(S2.3.2)}$$

The stoichiometry of these equations indicates that there will be one O atom consumed when one molecule of EO forms, while three O atoms will be consumed when one molecule of CO_2 and essentially one molecule of H_2O forms. Therefore, we can calculate the consumption rate of O atoms from the measured formation rates of EO and CO_2 (Eq. S2.3.3). The data points were collected every ~180 seconds, hence the moles of O atoms consumed during each interval can be calculated (Eq. S2.3.4).

$$- r_{O\ atom,\ total}\ [mol\cdot(g_{Ag}\cdot s)^{-1}] = - r_{O\ atom,\ EO} + - r_{O\ atom,\ CO2} = r_{EO} + 3\cdot(r_{CO2}) \quad (S2.3.3)$$

$$O\ atom\ [mol\cdot(g_{Ag})^{-1}] = - r_{O\ atom,\ total}\ [mol\cdot(g_{Ag}\cdot s)^{-1}]\cdot(180)\ [s] \quad (S2.3.4)$$

The bar graph in **Fig. S8A** shows the consumption of reactive oxygen atoms every 180 seconds and the accumulated consumption of O atoms (diamond). Approximately 36 $\mu mol\cdot(g_{Ag})^{-1}$ of oxygen atoms were consumed by reacting with ethylene within 60 minutes. We first examine the thickness of an Ag_2O layer consisting of 36 $\mu mol\cdot(g_{Ag})^{-1}$ of oxygen atoms on the surface of an 80 – 100 nm Ag nanoparticle. The molecular weight and density of Ag_2O are 231.735 $g\cdot mol^{-1}$ and 7.14 $g\cdot cm^{-3}$, respectively, while the molecular weight and density of Ag are 107.8682 $g\cdot mol^{-1}$and 10.49 $g\cdot cm^{-3}$, respectively. We then calculate the volume of this Ag_2O layer based on per cm^3 of Ag using the following equation:

$$36\ \mu mol\ O\cdot(g_{Ag})^{-1} = 36\ \mu mol\ Ag_2O\cdot(g_{Ag})^{-1} \quad (S2.3.5)$$
$$= \sim 0.0083\ g_{Ag2O}\cdot(g_{Ag})^{-1} = \sim 0.0012\ cm^3{}_{Ag2O}\cdot(0.095\ cm^3{}_{Ag})^{-1}$$
$$= \sim 0.013\ cm^3{}_{Ag2O}\cdot(cm^3{}_{Ag})^{-1} = \sim 1.3\ vol\%\ Ag_2O\cdot(Ag)^{-1}$$

Next, we assume that the core-shell nanoparticle is a sphere with a diameter of d, where the shell is Ag_2O, and the core is metallic Ag with a diameter of d_{Ag}. Therefore, the total volume of the core-shell nanoparticle is $\pi d^3/6$, and the volume of the metallic Ag core is $\pi d_{Ag}{}^3/6$. The thickness of the Ag_2O shell was then calculated using the following equations:

$$(\pi d^3/6 - \pi d_{Ag}{}^3/6) = (\pi d^3/6)\cdot(0.013) \quad (S2.3.6)$$
$$\text{Therefore, } (0.987)^{1/3}\cdot d = d_{Ag} \quad (S2.3.7)$$
$$\delta = d - d_{Ag}, \text{ where } \delta \text{ represents the thickness of the } Ag_2O \text{ shell} \quad (S2.3.8)$$

In this work, we used Ag nanoparticles with diameters around 80 – 100 nm. As a result, the surface Ag_2O layer that contains ~ 1.3 vol% $Ag_2O\cdot(Ag)^{-1}$ will have a thickness δ around 0.35 – 0.44 nm. This value is an absolute lower bound because it does not include surface/subsurface oxygen that did not react during the 60 minutes where oxygen was cutoff (i.e. the rates of production of oxygen containing species do not reach zero at the end of the 60 minutes).

Further, it should be noted that the actual reconstructed silver surface under reaction conditions forms a sub-stoichiometric $Ag_2O_{<1}$ oxide layer instead of Ag_2O. Therefore, the thickness of the layer containing reactive oxygen species must be larger than that calculated for a dense Ag_2O layer. Since it is difficult to determine the molecular weight for an $Ag_2O_{<1}$ structure, we adapted the molecular weight of Ag_2O and plotted the thickness of the Ag_2O layer as a function of Ag_2O density to mimic the oxide layer with different densities. **Fig. S8B** suggests that for an Ag_2O density that falls between 2 – 10 $g\cdot cm^{-3}$, δ would be less than 2 nm. Consequently, we estimate that the Ag surface binds reactive oxygen species to form a reconstructed surface layer that is less than 2 nm and likely less than 1 nm on the 80 – 100 nm Ag nanoparticles (**Fig. S8C**). The disordered nature of this thin layer was not captured by bulk characterization techniques (e.g., X-ray diffraction) here (and in previous studies) and may have been misinterpreted by surface characterization methods such as near-ambient X-ray photoelectron spectroscopy and vibrational spectroscopies in prior publications.

S2.4. Combination of O to regenerate O_2**

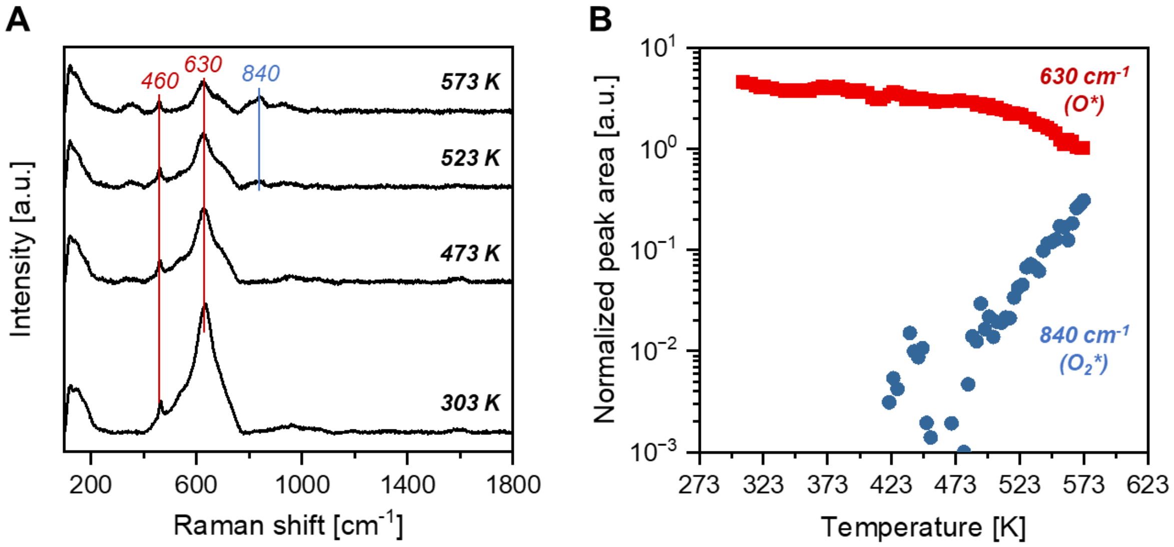

Fig. S9. *In situ* temperature-programmed Raman spectroscopy of initially reduced Ag catalysts under 101 kPa helium flow. (A) Raman spectra of Ag surface at different temperatures. (B) Changes in the peak area of the O* (630 cm^{-1}) and O_2* (840 cm^{-1}) features as a function of temperature. Ramp rate: 5 K·min^{-1}. Prior to this experiment, the Ag catalysts were pretreated oxidatively with 101 kPa O_2 flow at 673 K and then reductively with 101 kPa H_2 flow at 673 K (details of pretreatments described in the Experimental Section).

Section S3. Supporting Information for Steady-state Kinetic Measurements in the PFR System

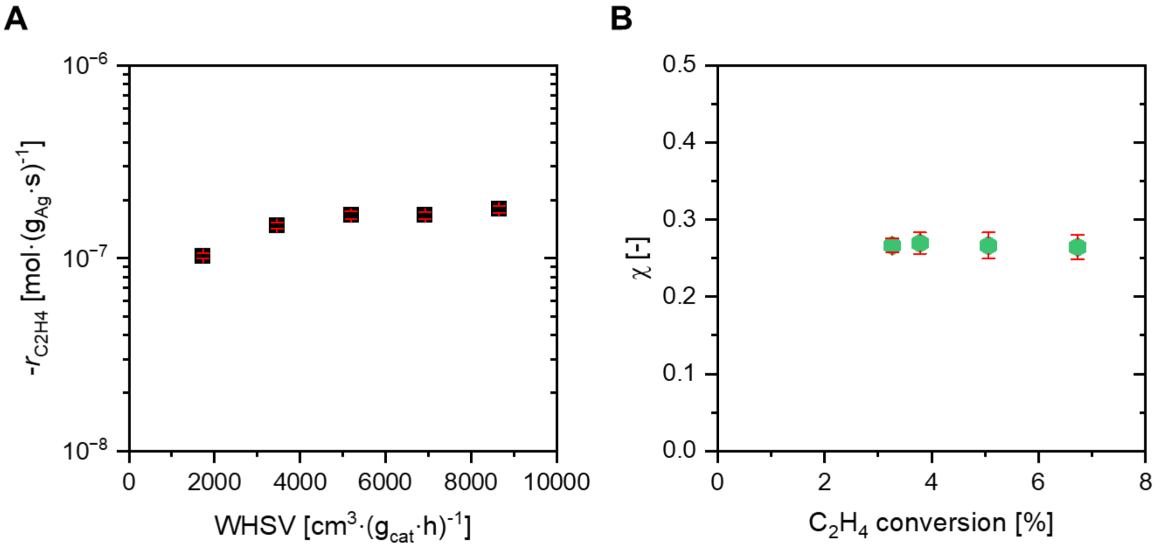

Fig. S10. Examination of mass transfer limitation and conversion effects on kinetics at 2 kPa C_2H_4, 20 kPa O_2, 523 K. (A) Dependency of ethylene reaction rates on weight hourly space velocity (WHSV), and (B) Change values of χ as a function of ethylene conversion.

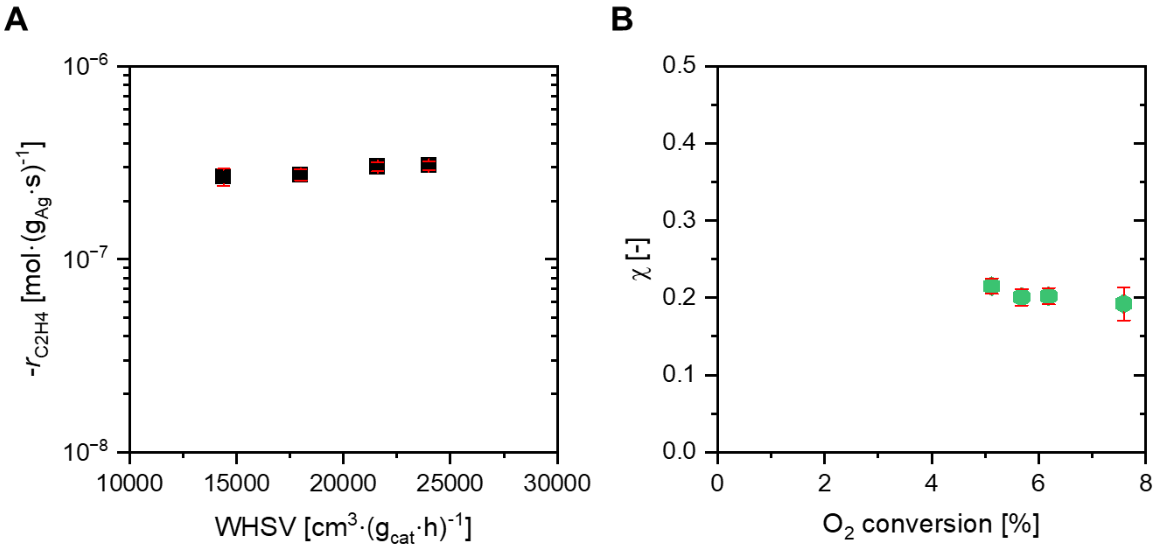

Fig. S11. Examination of mass transfer limitation and conversion effects on kinetics at 40 kPa C_2H_4, 2 kPa O_2, 523 K. (A) Dependency of ethylene reaction rates on weight hourly space velocity (WHSV), and (B) Change values of χ as a function of oxygen conversion.

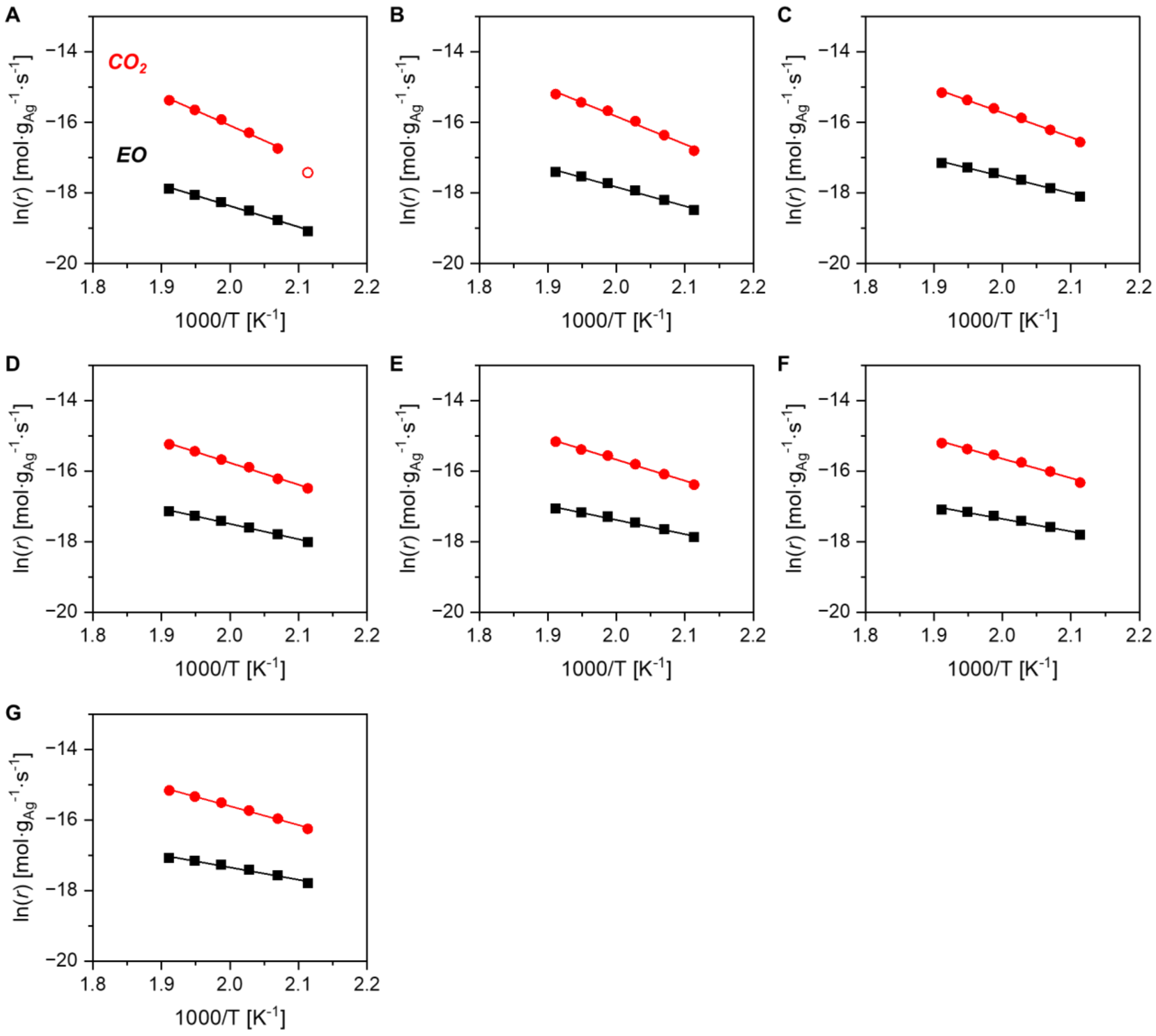

Fig. S12. Dependency of rates (*r*) on temperature (T) at 2 kPa C_2H_4 across a range of partial pressures of O_2. (A) 2 kPa O_2, (B) 8 kPa O_2, (C) 20 kPa O_2, (D) 40 kPa O_2, (E) 60 kPa O_2, (F) 70 kPa O_2, (G) 80 kPa O_2. Black square: EO, red circle: CO_2, T = 473 – 523 K (10 K/increment). All data points at the same P_{O2} were collected at identical weight hourly space velocity (WHSV) to eliminate possible artifacts caused by mass transport. The WHSV for different sets of experiments are: (A – D) ~7200, (E – F) ~9000, (G) ~8100 $cm^3 \cdot g_{cat}^{-1} \cdot h^{-1}$. At 2 kPa O_2, the formation rate of CO_2 at the lowest temperature (open point, 473 K) was excluded from linear fitting due to a low signal-to-noise ratio of the corresponding TCD peak. Apparent activation energies ($E_{a,\ app}$) derived from slopes of linear fit are listed in **Table S6** (*vide infra*).

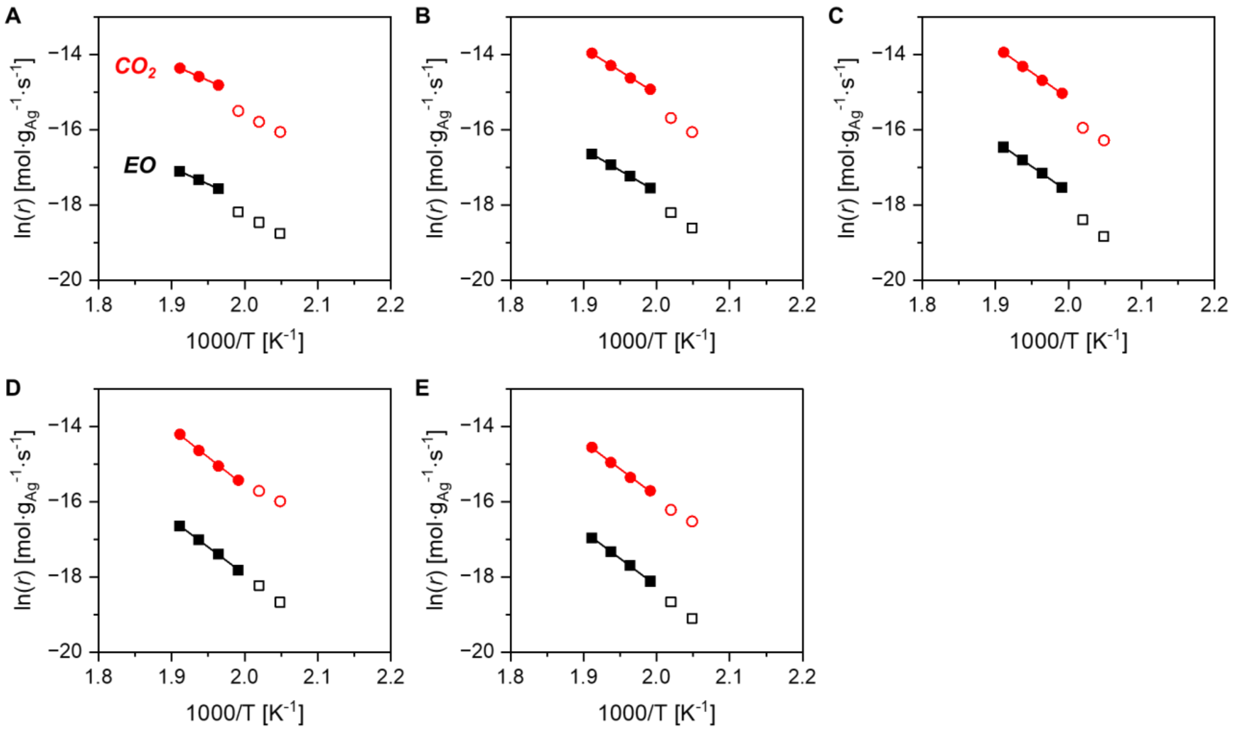

Fig. S13. Dependency of rates (r) on temperature (T) at 2 kPa O_2 across a range of partial pressures of C_2H_4. (A) 8 kPa C_2H_4, (B) 20 kPa C_2H_4, (C) 40 kPa C_2H_4, (D) 60 kPa C_2H_4, (E) 80 kPa C_2H_4. Black square: EO, red circle: CO_2, T = 488 – 523 K (7 K/increment). Open points indicate measurements that were taken at low weight hourly space velocity (WHSV ~7200 $cm^3 \cdot g_{cat}^{-1} \cdot h^{-1}$) with an intent to increase the conversion for better product detection, which were excluded from linear fitting due to possible artifacts of mass transportation. Solid points represent measurements taken at greater values of WHSV: (A – C) ~21600, (D) ~14400, and (E) ~10800 $cm^3 \cdot g_{cat}^{-1} \cdot h^{-1}$. Apparent activation energies ($E_{a,\,app}$) derived from slopes of linear fit are listed in **Table S6** (*vide infra*).

Table S6. Apparent activation energies ($E_{a, app}$) measured in this study

P_{O2} [kPa]	P_{C2H4} [kPa]	P_{O2}/P_{C2H4} [-]	$E_{a, app}$ (EO) [kJ·mol^{-1}]	$E_{a, app}$ (CO_2) [kJ·mol^{-1}]
2.02	80.8	0.025	120 ± 1.6	121 ± 3.6
2.02	60.6	0.033	121 ± 2.0	127 ± 3.9
2.02	40.4	0.050	112 ± 1.1	114 ± 2.5
2.02	20.2	0.10	94 ± 1.5	100 ± 2.5
2.02	8.08	0.25	73 ± 0.3	71 ± 0.2
2.02	2.02	1.0	46 ± 1.4	71 ± 3.8
8.08	2.02	4.0	45 ± 2.1	65 ± 3.6
20.2	2.02	10.0	40 ± 1.8	58 ± 2.1
40.4	2.02	20.0	36 ± 1.3	52 ± 1.5
60.6	2.02	30.0	34 ± 2.0	50 ± 1.7
70.7	2.02	35.0	29 ± 2.1	46 ± 2.4
80.8	2.02	40.0	29 ± 2.1	45 ± 1.6

Section S4. Reaction Paths

To determine if ethylene reacts differently with superoxo and peroxo dioxygen species, we computed the reaction energies and barriers on the superoxo- and peroxo-containing surfaces following two reaction mechanisms. First, the Langmuir-Hinshelwood (LH) mechanism proposed by Linic and Barteau, where ethylene reacts with O* to form an oxametallacycle (OMC) intermediate, which precedes both EO and AA products.[3-4] The LH mechanism was studied for EO reactions experimentally and computationally on different surface models, such as metallic Ag(111), Ag(110), Ag(100) at low and high coverages, and the p(4x4) reconstructed Ag surface.[5-8] Only recently have LH mechanisms with O_2* been considered on the Ag(111) and p(4x4) surfaces, with the emphasis on the oxygen content on different surfaces, rather than dioxygen speciation.[8] Second, we considered an Eley-Rideal (ER) mechanism, proposed by van Santen et al., where EO directly forms via reaction with O* and gas C_2H_4 on an $Ag_2O(001)$ slab.[9-11] Van Santen, however, did not consider reactions of ethylene with O_2* on $Ag_2O(001)$, ethylene epoxidation reactions with O* and O_2* were studied on metallic and reconstructed surfaces, however reactions with O_2* were not studied on surfaces with high content of subsurface oxygen. In the sections below we report the reaction energies corresponding to ethylene reactions with superoxo and peroxo O_2* species via both LH and ER mechanisms.

S4.1 ER mechanism

For direct reactions between C_2H_4 and O_2*, we generated an ensemble of structures with ethylene bonding with each O atom in the O_2*, where one carbon formed a bond with one of the O atoms while the other carbon was suspended above the surface. Subsequently, ethylene was rotated around the axis normal to the surface to make at least 5 initial guesses for each O atom. All structures were then optimized, with most of the relaxed structures remaining in the η^1-O_2-C_2H_4* (**Fig. S14A** and **S14B**) configuration. Our results show that the formation of η^1-O_2-C_2H_4* complex (**Fig. S15**, **Table S7** and **S8**), is endergonic for superoxo and peroxo-containing surface and therefore unlikely to form from direct adsorption of gas C_2H_4. Consequently, we consider an ER reaction with O_2* to be an unlikely pathway.

Next, we considered ER mechanisms where C_2H_4 directly reacts with O* to form EO, as proposed by van Santen and coworkers.[9-11] For each O* on the surface, we generated 5 or more initial guesses of ethylene adsorbing to O*, using the same procedure described above. All structures were optimized and most of them relaxed into an OMC* intermediate, where the second carbon atom bonded with the nearby surface Ag atom (See Fig. S19D). Only one initial structure on the superoxo surface resulted in a stable η^1-O_2-C_2H_4* intermediate (**Fig. S14C**) where carbon did not bond to Ag; however, the reaction was endothermic (**Table S7**). We were unable to locate the transition state for the reaction of C_2H_4(g) adsorbing to O* to form η^1-O-C_2H_4*, most likely due to the high energy of the product state and the fact that this intermediate product was higher in energy than all the OMC intermediates that formed spontaneously with geometry optimization. We also tried calculating the energy barrier where the initial state is the ethylene in the gas phase with O* on the surface, the transition state resembles η^1-O-C_2H_4* and the final state EO*. We were unable to locate the saddle point for both surfaces, with some of the interpolated images evolving into OMC intermediate. Taken together, our results uniformly show endergonic free energies to form η^1-O_2-C_2H_4* or η^1-O-C_2H_4* intermediates from gas C_2H_4 for superoxo/peroxo O_2*, and O*, respectively. Therefore, we do not consider the ER mechanism to be viable for EO formation on these surfaces.

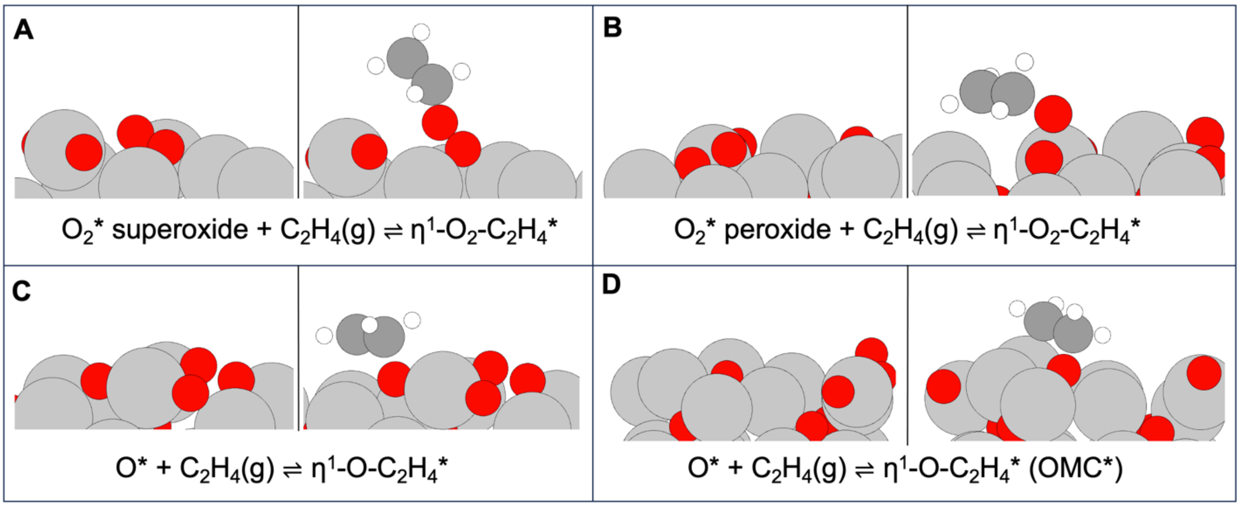

Fig. S14. Reaction intermediates for EO reaction with superoxo/peroxo O_2* and O* on both surfaces. Atom colors: O - red, Ag - light gray, C - dark gray, H - white.

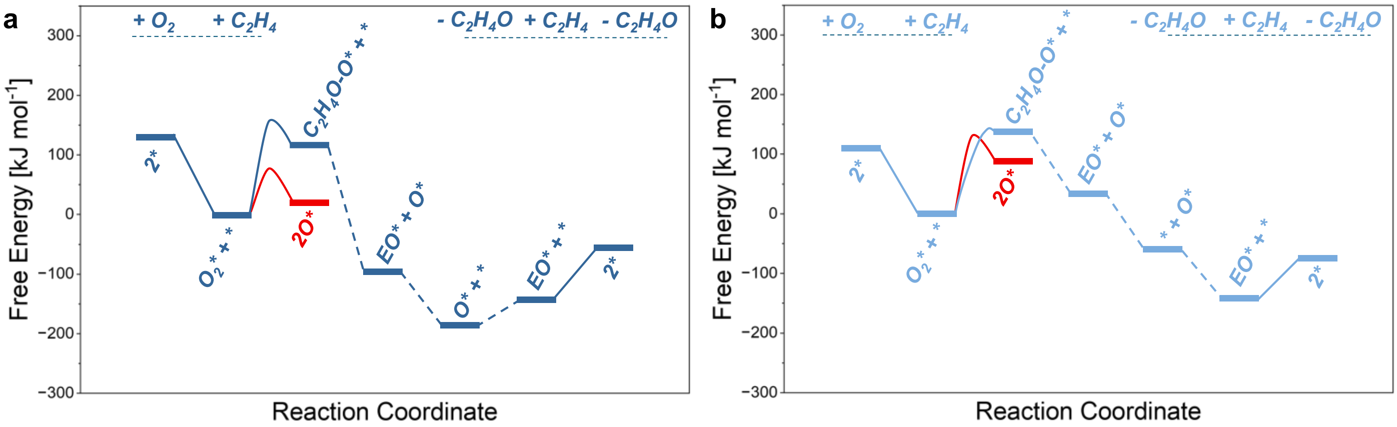

Fig. S15. Free energy coordinate of reactions among C_2H_4 and O_2 derived intermediates upon the surface that contains (a) peroxo and (b) superoxo species(O_2^-) with 2* representing (2111) and (1021) surfaces respectively. Free energies calculated at 523 K, 80 kPa O_2, 0.75 kPa C_2H_4, 0.15 kPa EO, 0.0375 kPa AA. Dashed lines indicate transition state calculations were not performed for the corresponding steps. Molecular formulas along the top indicate adsorption (+) and desorption (-) of gaseous species.

Table S7. Reaction energies for ER mechanism

Units (kJ/mol)	Superoxo	Peroxo
$O_2(g) + * \rightleftharpoons O_2*$	-213	-219
$O_2* \rightleftharpoons 2O*$	78	11
$C_2H_4(g) + O_2* \rightleftharpoons \eta^1\text{-}O_2\text{-}C_2H_4*$	35	37
$\eta^1\text{-}O_2\text{-}C_2H_4* \rightleftharpoons EO* + O*$	-120	-212
$EO* + O* \rightleftharpoons EO(g) + O*$	7	4
$C_2H_4(g) + O* \rightleftharpoons EO*$	-172	-53
$EO* \rightleftharpoons EO(g)$	36	47
$C_2H_4(g) + O* \rightleftharpoons \eta^1\text{-}O\text{-}C_2H_4*$	15	OMC formed

Table S8. Forward and reverse barrier for ER mechanism

	Superoxo		Peroxo	
Units (kJ/mol)	Forward	Reverse	Forward	Reverse
$C_2H_4(g) + O_2* \rightleftharpoons \eta^1\text{-}O_2\text{-}C_2H_4*$	137	0	150	34

S4.2. LH mechanism

On the superoxo and peroxo surfaces, we considered the adsorption of ethylene on each surface Ag atom in proximity of O_2* and assumed barrierless adsorption. For the reaction of C_2H_4* with O_2* to generate an η^2-O_2-C_2H_4* intermediate (see product in **Fig. S19A**) we considered at least 6 initial guess structures of ethylene binding to each of the oxygen atoms in O_2* and the nearby Ag atoms. For further reactions from η^2-O_2-C_2H_4* we tested three possible outcomes, η^2-O_2-C_2H_4* going to EO* + O*, AA* + O*, and η^2-O-C_2H_4* + O* (ethylene assisted O_2* dissociation). We were unable to locate a transition state for the η^2-O_2-C_2H_4* going directly to EO* or AA* on either surface. Ethylene assisted O_2* dissociation to form η^2-O-C_2H_4* + O* was exergonic for peroxo surface (**Fig. 4b** in Main Text and **Table S9**), with a free energy barrier of 11 kJ/mol and it was exergonic for the superoxo surface (**Fig. S16** in Main Text and **Table S9**), with a free energy barrier of 105 kJ/mol, therefore we do not consider this reaction viable on superoxo surface. More favorable reaction energies and barriers on the peroxo surface to form η^2-O-C_2H_4* + O* is likely due to higher O-O distance in η^2-O_2-C_2H_4*, caused by higher charge density on the O atoms (see **Fig. S3** and **Tables S4** and **S5**) for comparison of charges in O atoms in η^2-O_2-C_2H_4* intermediate on superoxo and peroxo surfaces). Furthermore, we considered direct dissociation of O_2* and subsequent reactions with C_2H_4* to form η^2-O-C_2H_4* + O*, maximum free energy for η^2-O-C_2H_4* through C_2H_4* assisted O_2 dissociation and direct dissociation pathway were similar on peroxo and superoxo surfaces (**Fig. 4b** in Main text and **Fig. S16**), highlighting the competitive nature of the two pathways. When generating TS to form AA from η^2-O-C_2H_4* + O* or η^2-O-C_2H_4* (described in the paragraph below), we considered the transfer of both H atoms from the C atom bound to O, to the C atom bound to Ag. The barriers to forming EO* and AA* on the peroxo surface were low and similar to one another, showing that O_2^{2-} species can be a precursor to EO generation.

After one O atom from the O_2* has reacted and left the surface as EO or AA species, we considered C_2H_4 reactions with the remaining O* atoms on the peroxo and superoxo surfaces. We considered two pathways for C_2H_4 reactions – C_2H_4 adsorption on an Ag atom near an O* species to form C_2H_4*, and C_2H_4 from the gas phase directly reacting with O* to form η^2-O-C_2H_4*, see Fig. S19E. To generate η^2-O-C_2H_4* we made 3 initial guesses where one carbon in C_2H_4 binds to Ag atom and the second carbon binds to O* (see Fig. S19E, product structure). On the superoxo surface, we only found the transition state for C_2H_4(g) directly reacting with O* to form η^2-O-C_2H_4*, while on the peroxo surface we found both transition states for C_2H_4* + O* and C_2H_4(g) + O* to form η^2-O-C_2H_4*, with the latter having lower barrier (see **Table S10**). Following the formation of η^2-O-C_2H_4*, we considered EO* and AA* formation on the peroxo surface and found that both barriers were higher than the respective barriers from the η^2-O-C_2H_4* + O* in the earlier part of the chemical cycle.

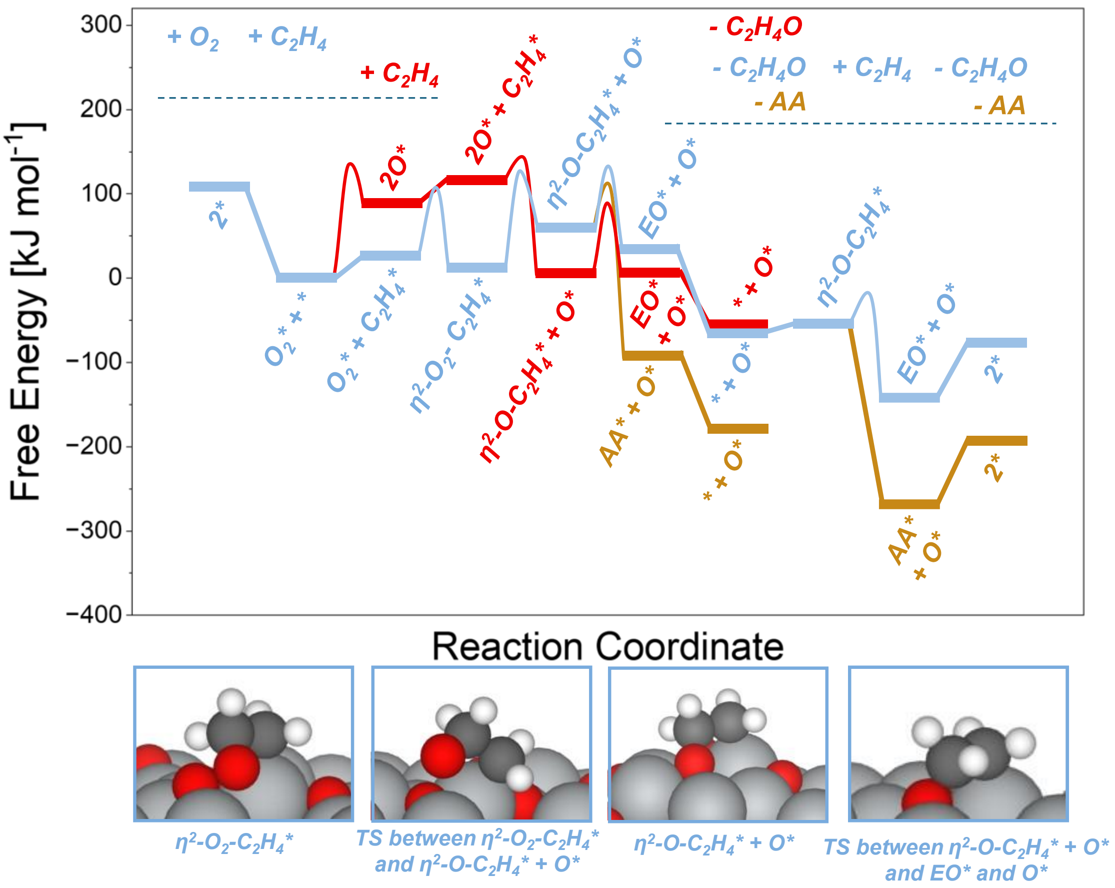

Fig. S16. Free energy coordinate of reactions among C_2H_4 and O_2 derived intermediates upon the surface that contains superoxo species(O_2^-). Free energies calculated at 523 K, 80 kPa O_2, 0.75 kPa C_2H_4, 0.15 kPa EO, 0.0375 kPa AA. Values in parenthesis indicate intrinsic activation energies. 2* represents (1021 surface). Molecular formulas along the top indicate adsorption (+) and desorption (-) of gaseous species. Atom colors: O - red, Ag - light gray, C - dark gray, H - white.

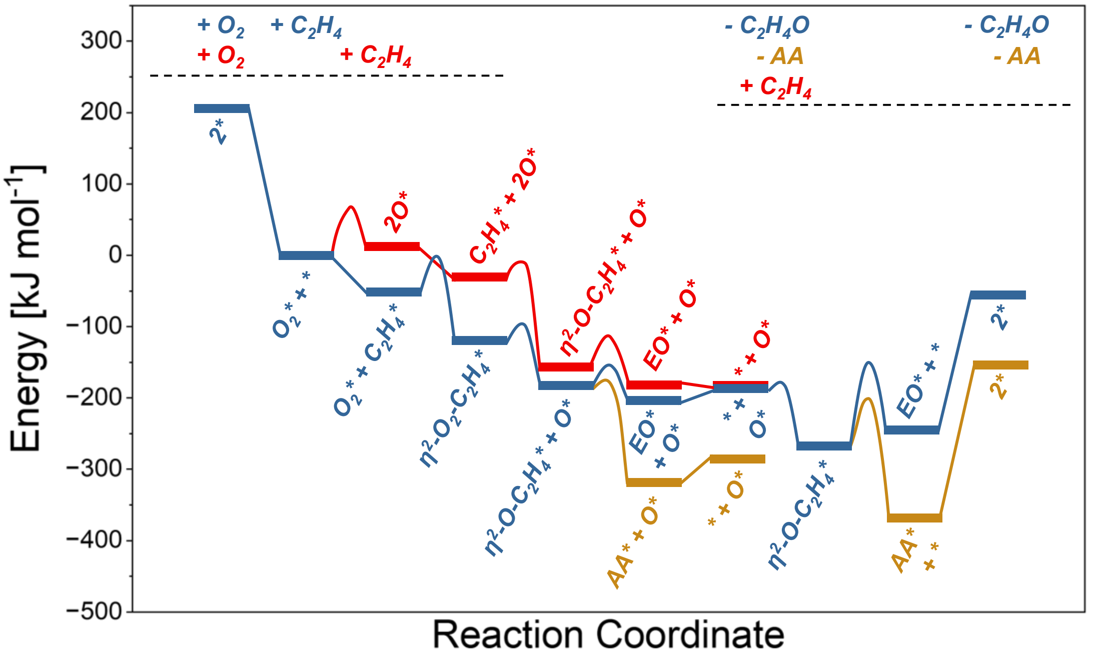

Fig. S17. Reaction energy coordinate of reactions among C_2H_4 and O_2 derived intermediates upon the that contains peroxo species(O_2^-). Values in parenthesis indicate intrinsic activation energies. 2* represents (2111 surface). Molecular formulas along the top indicate adsorption (+) and desorption (-) of gaseous species.

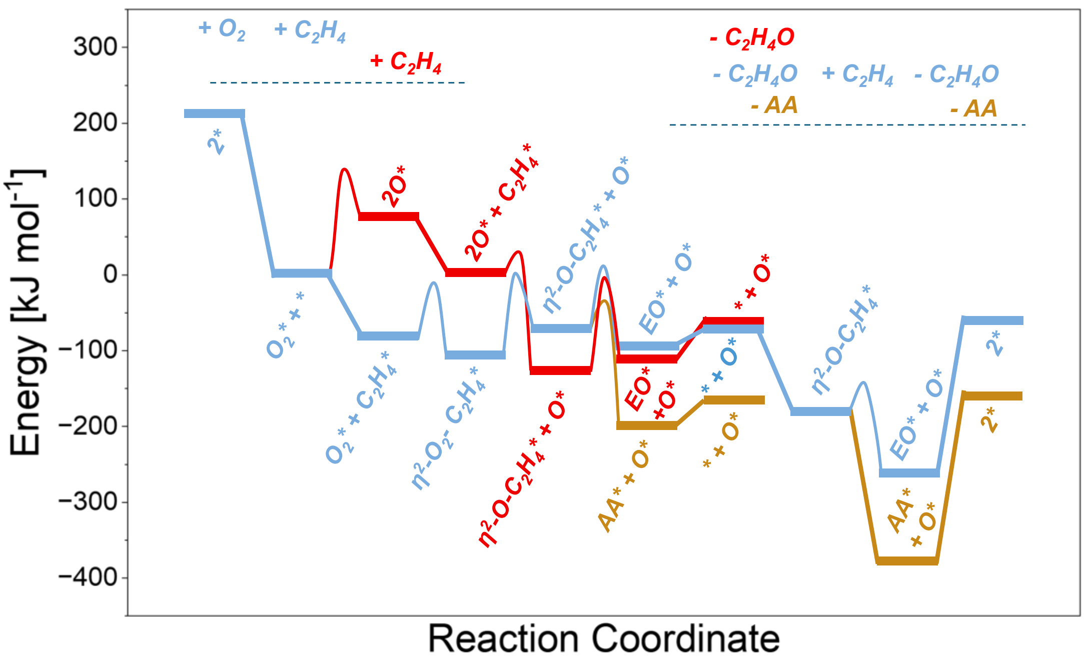

Fig. S18. Reaction energy coordinate of reactions among C_2H_4 and O_2 derived intermediates upon the that contains superoxo species(O_2^-). Values in parenthesis indicate intrinsic activation energies. 2* represents (1021 surface). Molecular formulas along the top indicate adsorption (+) and desorption (-) of gaseous species.

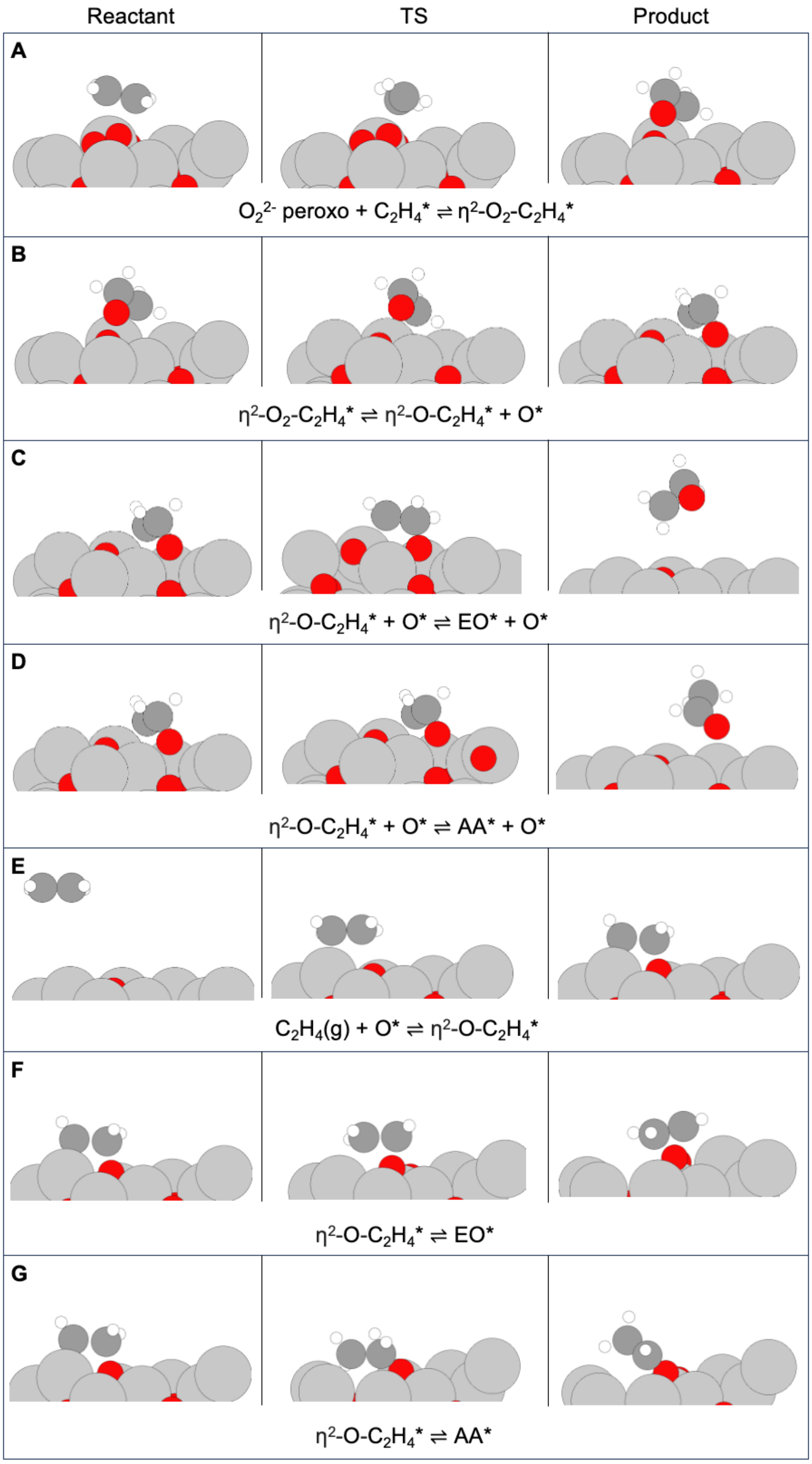

Fig. S19. Reaction intermediates for EO/AA reactions with peroxo surface with O_2^{2-} species. Atom colors: O - red, Ag - light gray, C - dark gray, H - white.

Table S9. Reaction energies and free energies for LH mechanism on superoxo and peroxo surfaces. Free energies calculated at 523 K, 80 kPa O_2, 0.75 kPa C_2H_4, 0.15 kPa EO, 0.0375 kPa AA.

		Reaction energy		Free energy	
**Reaction pathway**	**Units (kJ/mol)**	**Superoxo**	**Peroxo**	**Superoxo**	**Peroxo**
C_2H_4* assisted O_2* dissociation pathway	O_2(g) + * ⇌O_2*	-213	-219	-109	-129
	C_2H_4(g) + O_2* ⇌ C_2H_4* +O_2*	-82	11	26	44
	C_2H_4* + O_2* ⇌ η^2-O_2-C_2H_4*	-25	-52	-14	-44
	η^2-O_2-C_2H_4* ⇌ η^2-O-C_2H_4* + O*	36	-68	46	-55
	η^2-O-C_2H_4* + O* ⇌ EO* + O*	-22	-64	-25	-42
	EO* + O* ⇌ EO(g) + O*	27	-20	-94	-89
	η^2-O-C_2H_4* + O* ⇌ AA* + O*	-131	18	-152	-168
	AA* + O* ⇌ AA(g) + O*	35	-137	-86	-81
	C_2H_4(g) + O* ⇌ η^2-O-C_2H_4*	-115	35	5	32
	η^2-O-C_2H_4* ⇌ EO*	-82	-82	-86	9
	EO* ⇌ EO(g)	202	21	66	88
	η^2-O-C_2H_4* ⇌ AA*	-200	191	-213	-126
	AA* ⇌ AA(g)	219	-103	74	104
Direct O_2 dissociation pathway	O_2(g) + * ⇌O_2*	-213	-219	-88	-109
	O_2* ⇌ 2O*	78	11	89	21
	C_2H_4(g) + 2O* ⇌ C_2H_4*+2O*	-76	-42	27	55
	C_2H_4*+2O* ⇌ η^2-O-C_2H_4*+O*	-129	-127	-111	-109
	η^2-O-C_2H_4* + O* ⇌ EO* + O*	15	-26	2	-44
	EO* + O* ⇌ EO(g) + O*	50	-2	-60	-111

Table S10. Forward and reverse barriers for LH mechanism on superoxo and peroxo surfaces

		**Superoxo**		**Peroxo**	
**Reaction pathway**	**Units (kJ/mol)**	Forward	Reverse	Forward	Reverse
C_2H_4* assisted O_2* dissociation pathway	C_2H_4* +O_2* ⇌ η^2-O_2-C_2H_4*	66	91	44	112
	η^2-O_2-C_2H_4* ⇌ η^2-O-C_2H_4* +O*	98	62	13	77
	η^2-O-C_2H_4* + O* ⇌ EO* + O*	75	97	23	43
	η^2-O-C_2H_4* + O* ⇌ AA* + O*	63	194	1	139
	C_2H_4(g) +O* ⇌ η^2-O-C_2H_4*	0	115	0	75
	$C_2H_4^*$ +O* ⇌ η^2-O-C_2H_4*	-	-	31	76
	η^2-O-C_2H_4* ⇌ EO*	29	110	108	87
	η^2-O-C_2H_4* ⇌ AA*	0	200	67	170
Direct O_2* dissociation pathway	O_2* ⇌ 2O*	118	41	64	53
	C_2H_4*+2O* ⇌ η^2-O-C_2H_4*+O*	17	146	14	141
	η^2-O-C_2H_4* + O* ⇌ EO* + O*	80	64	38	65

Table S11. Free energy forward and reverse barriers for LH mechanism on superoxo and peroxo surfaces calculated at 523 K, 80 kPa O_2, 0.75 kPa C_2H_4, 0.15 kPa EO, 0.0375 kPa AA.

		**Superoxo**		**Peroxo**	
	**Units (kJ/mol)**	Forward	Reverse	Forward	Reverse
C_2H_4* assisted O_2* dissociation pathway	C_2H_4* +O_2* ⇌ η^2-O_2-C_2H_4*	72	86	55	99
	η^2-O_2-C_2H_4* ⇌ η^2-O-C_2H_4* +O*	105	59	11	66
	η^2-O-C_2H_4* + O* ⇌ EO* + O*	66	91	28	70
	η^2-O-C_2H_4* + O* ⇌ AA* + O*	48	200	1	169
	C_2H_4(g) +O* ⇌ η^2-O-C_2H_4*	0	5	99	67
	η^2-O-C_2H_4* ⇌ EO*	29	115	95	86
	η^2-O-C_2H_4* ⇌ AA*	0	213	48	175
Direct O_2* dissociation pathway	O_2* ⇌ 2O*	127	38	69	48
	C_2H_4*+2O* ⇌ η^2-O-C_2H_4*+O*	19	131	17	126
	η^2-O-C_2H_4* + O* ⇌ EO* + O*	74	72	37	81

Table S12: Reaction energies and free energies for O_2* recombination at higher total O* coverage (at the beginning of reaction/free energy diagram) and lower total O* coverage (after EO desorption) on superoxo and peroxo surface. Free energies calculated at 523 K, 80 kPa O_2, 0.75 kPa C_2H_4, 0.15 kPa EO, 0.0375 kPa AA.

		**Reaction energy**		**Free energy**	
	**Units (kJ/mol)**	**Superoxo**	**Peroxo**	**Superoxo**	**Peroxo**
High total O* coverage	$2O^* \rightleftharpoons O_2^*$	-78	-11	-89	-21
Lower total O* coverage	$2O^* \rightleftharpoons O_2^*$	122	159	121	150

Section S5. Derivation of Analytical Rate Expressions

S5.1. Ethylene activation via surface reactions with O and O₂**

We simplified the reaction steps by eliminating surface intermediates that occupy two sites (i.e., η^2-O-C_2H_4 and η^2-O_2-C_2H_4), and we neglected surface EO intermediate (C_2H_4O*) by assuming that EO desorbs immediately after formation. In addition, we assume that the surface acetaldehyde intermediate (CH_3CHO*) transforms to CO_2 and H_2O via sequential oxidation steps with O* that involve CH_2O* and CO* intermediates.

Step	Equation	Rate constant
1	$O_{2(g)} + 2^* \rightleftharpoons O_2^* + ^*$	k_1, k_{-1}
2	$O_2^* + 2^* \rightleftharpoons 2O^* + ^*$	k_2, k_{-2}
3	$C_2H_{4(g)} + 2^* \rightleftharpoons C_2H_4^* + ^*$	k_3, k_{-3}
4	$C_2H_4^* + O^* \rightarrow C_2H_4O_{(g)} + 2^*$	k_4
5	$C_2H_4^* + O^* \rightarrow CH_3CHO^* + ^*$	k_5
6	$C_2H_4^* + O_2^* \rightarrow C_2H_4O_{(g)} + O^* + ^*$	k_6
7	$C_2H_4^* + O_2^* \rightarrow CH_3CHO^* + O^*$	k_7
8	$CH_3CHO^* + O^* \rightarrow 2CH_2O^*$	k_8
9	$CH_2O^* + O^* \rightarrow H_2O_{(g)} + CO^* + ^*$	k_9
10	$CO^* + O^* \rightarrow CO_{2(g)} + 2^*$	k_{10}

Therefore, performing site balance based on the reaction steps gives:

$$[L] = [^*] + [O^*] + [O_2^*] + [C_2H_4^*] + [CH_3CHO^*] + [CH_2O^*] + [CO^*] \quad (S5.1)$$

Where [L] represents the total number of sites on the silver surface, [*] denotes unoccupied Ag sites, and [X*] denotes adsorbates bound to such sites.

By applying quasi-equilibrium for the adsorption and desorption of O_2 (step1) and C_2H_4 (step 3), we have:

$$k_1 P_{O_2} \frac{[^*]^2}{[L]} \cong k_{-1} \frac{[O_2^*]\cdot[^*]}{[L]} \quad (S5.2)$$

$$k_3 P_{C_2H_4} \frac{[^*]^2}{[L]} \cong k_{-3} \frac{[C_2H_4^*]\cdot[^*]}{[L]} \quad (S5.3)$$

Solving equations S5.2 and S5.3 gives:

$$[O_2^*] = K_1 P_{O_2}[^*], \text{ where } K_1 = k_1/k_{-1} \quad (S5.4)$$

$$[C_2H_4^*] = K_3 P_{C_2H_4}[^*], \text{ where } K_3 = k_3/k_{-3} \quad (S5.5)$$

Applying pseudo steady-state hypothesis for [O*], [CH_3CHO*], [CH_2O*], and [CO*] leads to:

$$\frac{d[O^*]}{dt} \cong 0 = 2k_2\frac{[O_2^*]\cdot[^*]}{[L]} - 2k_{-2}\frac{[O^*]^2}{[L]} - (k_4 + k_5)\frac{[C_2H_4^*]\cdot[O^*]}{[L]} + (k_6 + k_7)\frac{[C_2H_4^*]\cdot[O_2^*]}{[L]} - k_8\frac{[CH_3CHO^*]\cdot[O^*]}{[L]} - k_9\frac{[CH_2O^*]\cdot[O^*]}{[L]} - k_{10}\frac{[CO^*]\cdot[O^*]}{[L]} \quad (S5.6)$$

$$\frac{d[CH_3CHO^*]}{dt} \cong 0 = k_5\frac{[C_2H_4^*]\cdot[O^*]}{[L]} + k_7\frac{[C_2H_4^*]\cdot[O_2^*]}{[L]} - k_8\frac{[CH_3CHO^*]\cdot[O^*]}{[L]} \quad (S5.7)$$

$$\frac{d[CH_2O^*]}{dt} \cong 0 = 2k_8\frac{[CH_3CHO^*]\cdot[O^*]}{[L]} - k_9\frac{[CH_2O^*]\cdot[O^*]}{[L]} \quad (S5.8)$$

$$\frac{d[CO^*]}{dt} \cong 0 = k_9\frac{[CH_2O^*]\cdot[O^*]}{[L]} - k_{10}\frac{[CO^*]\cdot[O^*]}{[L]} \quad (S5.9)$$

Therefore,

$$[CH_3CHO^*]\cdot[O^*] = \frac{k_5}{k_8}K_3P_{C_2H_4}[^*]\cdot[O^*] + \frac{k_7}{k_8}K_3P_{C_2H_4}[^*]\cdot K_1P_{O_2}[^*] \quad (S5.10)$$

$$[CH_2O^*]\cdot[O^*] = \frac{2k_8}{k_9}[CH_3CHO^*]\cdot[O^*] \quad (S5.11)$$

$$[CO^*]\cdot[O^*] = \frac{k_9}{k_{10}}[CH_2O^*]\cdot[O^*] = \frac{2k_8}{k_{10}}[CH_3CHO^*]\cdot[O^*] \quad (S5.12)$$

Substituting equations S5.10, S5.11, and S5.12 to S5.6 gives:

$$\frac{d[O^*]}{dt} \cong 0 = 2k_2K_1P_{O_2}[^*]^2 - 2k_{-2}[O^*]^2 - (k_4 + k_5)K_3P_{C_2H_4}[^*]\cdot[O^*] + (k_6 + k_7)K_1P_{O_2}K_3P_{C_2H_4}[^*]^2 - 5k_8(\frac{k_5}{k_8}K_3P_{C_2H_4}[^*]\cdot[O^*] + \frac{k_7}{k_8}K_1P_{O_2}K_3P_{C_2H_4}[^*]^2) \quad (S5.13)$$

Equation S5.13 can be transformed to:

$$2k_{-2}[O^*]^2 + K_3P_{C_2H_4}(k_4 + 6k_5)[O^*]\cdot[^*] - K_1P_{O_2}\{ 2k_2 + K_3P_{C_2H_4}(k_6 - 4k_7)\}[^*]^2 = 0 \quad (S5.14)$$

Solving equation S5.14 leads to [O*] = β[*], where β represents:

$$\beta = -\frac{K_3P_{C_2H_4}(k_4 + 6k_5)}{4k_{-2}} + \sqrt{\left\{\frac{K_3P_{C_2H_4}(k_4 + 6k_5)}{4k_{-2}}\right\}^2 + \frac{K_1P_{O_2}\left\{2k_2 + K_3P_{C_2H_4}(k_6 - 4k_7)\right\}}{2k_{-2}}} \quad (S5.15)$$

Therefore, equations S5.10, S5.11, and S5.12 can be expressed as:

$$[CH_3CHO^*] = \frac{K_3P_{C_2H_4}(k_5\beta + k_7K_1P_{O_2})}{k_8\beta} \quad (S5.16)$$

$$[CH_2O^*] = \frac{2k_8}{k_9}[CH_3CHO^*] = \frac{2K_3P_{C_2H_4}(k_5\beta + k_7K_1P_{O_2})}{k_9\beta} \quad (S5.17)$$

$$[CO^*] = \frac{2k_8}{k_{10}}[CH_3CHO^*] = \frac{2K_3P_{C_2H_4}(k_5\beta + k_7K_1P_{O_2})}{k_{10}\beta} \quad (S5.18)$$

Then, we can rewrite the site balance (S5.1) as follows by defining [L] = α[*], where α represents:

$$\alpha = 1 + \beta + K_1P_{O_2} + K_3P_{C_2H_4} + \frac{K_3P_{C_2H_4}(k_5\beta + k_7K_1P_{O_2})}{\beta}(\frac{1}{k_8} + \frac{2}{k_9} + \frac{2}{k_{10}}) \quad (S5.19)$$

Finally, the analytical expressions of rates and χ can be written in the following forms:

$$\frac{r_{EO}}{[L]} = k_4 \frac{[C_2H_4*]\cdot[O*]}{[L]^2} + k_6 \frac{[C_2H_4*]\cdot[O_2*]}{[L]^2} = \frac{K_3 P_{C_2H_4}(k_4\beta + k_6 K_1 P_{O_2})}{\alpha^2} \quad (S5.20)$$

$$\frac{r_{CO_2}/2}{[L]} = k_{10} \frac{[CO*]\cdot[O*]}{2[L]^2} = \frac{K_3 P_{C_2H_4}(k_5\beta + k_7 K_1 P_{O_2})}{\alpha^2} \quad (S5.21)$$

$$\chi = \frac{r_{EO}}{r_{CO_2}/2} = \frac{k_4\beta + k_6 K_1 P_{O_2}}{k_5\beta + k_7 K_1 P_{O_2}} \quad (S5.22)$$

These expressions suggest that when ethylene is activated by both O* and O_2* to form EO or AA/CO_2, χ would depend on the coverages of atomic and diatomic oxygens species. Steady-state and transient *operando* Raman experiments indicate that χ increases with the coverage of O_2^{2-} species, which increases as P_{O2} increases. Therefore, an increasing value of χ that we observed experimentally at a range of P_{O2}/P_{C2H4} from 0.25 to 10 suggests that while both ethylene activation is in a regime involving both O* and O_2^{2-}, the contribution of O_2^{2-} on the activation of ethylene gradually becomes significant as opposed to the contribution of O*.

S5.2. Ethylene activation via surface reactions with only O*

Step	Equation	Rate constant
1	$O_{2(g)} + 2* \rightleftharpoons O_2* + *$	k_1, k_{-1}
2	$O_2* + 2* \rightleftharpoons 2O* + *$	k_2, k_{-2}
3	$C_2H_{4(g)} + 2* \rightleftharpoons C_2H_4* + *$	k_3, k_{-3}
4	$C_2H_4* + O* \rightarrow C_2H_4O_{(g)} + 2*$	k_4
5	$C_2H_4* + O* \rightarrow CH_3CHO* + *$	k_5
8	$CH_3CHO* + O* \rightarrow 2CH_2O*$	k_8
9	$CH_2O* + O* \rightarrow H_2O_{(g)} + CO* + *$	k_9
10	$CO* + O* \rightarrow CO_{2(g)} + 2*$	k_{10}

Performing site balance based on the reaction steps also leads to equation S5.1:

$$[L] = [*] + [O*] + [O_2*] + [C_2H_4*] + [CH_3CHO*] + [CH_2O*] + [CO*] \quad (S5.1)$$

By assuming quasi-equilibrium for the adsorption and desorption of O_2 (step1) and C_2H_4 (step 3), and by applying pseudo steady-state hypothesis for [O*], [CH_3CHO*], [CH_2O*], and [CO*], we have:

$$\frac{d[O*]}{dt} \cong 0 = 2k_2\frac{[O_2*]\cdot[*]}{[L]} - 2k_{-2}\frac{[O*]^2}{[L]} - (k_4 + k_5)\frac{[C_2H_4*]\cdot[O*]}{[L]} - k_8\frac{[CH_3CHO*]\cdot[O*]}{[L]} - k_9\frac{[CH_2O*]\cdot[O*]}{[L]} - k_{10}\frac{[CO*]\cdot[O*]}{[L]} \quad (S5.23)$$

$$\frac{d[CH_3CHO*]}{dt} \cong 0 = k_5\frac{[C_2H_4*]\cdot[O*]}{[L]} - k_8\frac{[CH_3CHO*]\cdot[O*]}{[L]} \quad (S5.24)$$

$$\frac{d[CH_2O*]}{dt} \cong 0 = 2k_8\frac{[CH_3CHO*]\cdot[O*]}{[L]} - k_9\frac{[CH_2O*]\cdot[O*]}{[L]} \quad (S5.25)$$

$$\frac{d[CO*]}{dt} \cong 0 = k_9\frac{[CH_2O*]\cdot[O*]}{[L]} - k_{10}\frac{[CO*]\cdot[O*]}{[L]} \quad (S5.26)$$

Therefore,

$$[CH_3CHO*]\cdot[O*] = \frac{k_5}{k_8}K_3P_{C_2H_4}[*]\cdot[O*] \quad (S5.27)$$

$$[CH_2O*]\cdot[O*] = \frac{2k_8}{k_9}[CH_3CHO*]\cdot[O*] = \frac{2k_5}{k_9}K_3P_{C_2H_4}[*]\cdot[O*] \quad (S5.28)$$

$$[CO*]\cdot[O*] = \frac{k_9}{k_{10}}[CH_2O*]\cdot[O*] = \frac{2k_5}{k_{10}}K_3P_{C_2H_4}[*]\cdot[O*] \quad (S5.29)$$

Substituting equations S5.27, S5.28, and S5.29 to S5.23 gives:

$$\frac{d[O*]}{dt} \cong 0 = 2k_2K_1P_{O_2}[*]^2 - 2k_{-2}[O*]^2 - (k_4 + 6k_5)K_3P_{C_2H_4}[*]\cdot[O*] \quad (S5.30)$$

Equation S5.30 can be transformed to:

$$2k_{-2}[O*]^2 + K_3P_{C_2H_4}(k_4 + 6k_5)[O*]\cdot[*] - K_1P_{O_2}(2k_2)[*]^2 = 0 \quad (S5.31)$$

Solving equation S5.31 leads to [O*] = β[*], where β represents:

$$\beta = -\frac{K_3P_{C_2H_4}(k_4 + 6k_5)}{4k_{-2}} + \sqrt{\left\{\frac{K_3P_{C_2H_4}(k_4 + 6k_5)}{4k_{-2}}\right\}^2 + \frac{2k_2K_1P_{O_2}}{2k_{-2}}} \quad (S5.32)$$

Therefore, equations S5.27, S5.28, and S5.29 can be expressed as:

$$[CH_3CHO^*] = \frac{k_5K_3P_{C_2H_4}}{k_8} \quad (S5.33)$$

$$[CH_2O^*] = \frac{2k_8}{k_9}[CH_3CHO^*] = \frac{2k_5K_3P_{C_2H_4}}{k_9} \quad (S5.34)$$

$$[CO^*] = \frac{2k_8}{k_{10}}[CH_3CHO^*] = \frac{2k_5K_3P_{C_2H_4}}{k_{10}} \quad (S5.35)$$

Then, we can rewrite the site balance (S5.1) as follows by defining [L] = α[*], where α represents:

$$\alpha = 1 + \beta + K_1P_{O_2} + K_3P_{C_2H_4} + k_5K_3P_{C_2H_4}\left(\frac{1}{k_8} + \frac{2}{k_9} + \frac{2}{k_{10}}\right) \quad (S5.36)$$

Finally, the analytical expressions of rates and χ can be written in the following forms:

$$\frac{r_{EO}}{[L]} = k_4\frac{[C_2H_4^*]\cdot[O^*]}{[L]^2} = \frac{K_3P_{C_2H_4}(k_4\beta)}{\alpha^2} \quad (S5.37)$$

$$\frac{r_{CO_2}/2}{[L]} = k_{10}\frac{[CO^*]\cdot[O^*]}{2[L]^2} = \frac{K_3P_{C_2H_4}(k_5\beta)}{\alpha^2} \quad (S5.38)$$

$$\chi = \frac{r_{EO}}{r_{CO_2}/2} = \frac{k_4}{k_5} \quad (S5.39)$$

These expressions suggest that when ethylene is activated by only O* to form EO or AA/CO_2, χ would be a constant that is equal to k_4/k_5, indicating that the selectivity would likely be determined by the barriers of reaction step 4 and 5. This scenario agrees with our experimental observation at the low P_{O2}/P_{C2H4} regime (i.e., P_{O2}/P_{C2H4} = 0.025 ~ 0.05), where coverages of surface O_2^{2-} species are the lowest and χ is a constant value (~0.35).

S5.3. Ethylene activation via surface reactions with only O_2*

Step	Equation	Rate constant
1	$O_{2(g)} + 2^* \rightleftharpoons O_2^* + ^*$	k_1, k_{-1}
2	$O_2^* + 2^* \rightleftharpoons 2O^* + ^*$	k_2, k_{-2}
3	$C_2H_{4(g)} + 2^* \rightleftharpoons C_2H_4^* + ^*$	k_3, k_{-3}
6	$C_2H_4^* + O_2^* \rightarrow C_2H_4O_{(g)} + O^* + ^*$	k_6
7	$C_2H_4^* + O_2^* \rightarrow CH_3CHO^* + O^*$	k_7
8	$CH_3CHO^* + O^* \rightarrow 2CH_2O^*$	k_8
9	$CH_2O^* + O^* \rightarrow H_2O_{(g)} + CO^* + ^*$	k_9
10	$CO^* + O^* \rightarrow CO_{2(g)} + 2^*$	k_{10}

Performing site balance based on the reaction steps also leads to equation S5.1:

$$[L] = [^*] + [O^*] + [O_2^*] + [C_2H_4^*] + [CH_3CHO^*] + [CH_2O^*] + [CO^*] \qquad (S5.1)$$

By assuming quasi-equilibrium for the adsorption and desorption of O_2 (step1) and C_2H_4 (step 3), and by applying pseudo steady-state hypothesis for [O*], [CH_3CHO*], [CH_2O*], and [CO*], we have:

$$\frac{d[O^*]}{dt} \cong 0 = 2k_2\frac{[O_2^*]\cdot[^*]}{[L]} - 2k_{-2}\frac{[O^*]^2}{[L]} + (k_6 + k_7)\frac{[C_2H_4^*]\cdot[O_2^*]}{[L]} - k_8\frac{[CH_3CHO^*]\cdot[O^*]}{[L]} - k_9\frac{[CH_2O^*]\cdot[O^*]}{[L]} - k_{10}\frac{[CO^*]\cdot[O^*]}{[L]} \qquad (S5.40)$$

$$\frac{d[CH_3CHO^*]}{dt} \cong 0 = k_7\frac{[C_2H_4^*]\cdot[O_2^*]}{[L]} - k_8\frac{[CH_3CHO^*]\cdot[O^*]}{[L]} \qquad (S5.41)$$

$$\frac{d[CH_2O^*]}{dt} \cong 0 = 2k_8\frac{[CH_3CHO^*]\cdot[O^*]}{[L]} - k_9\frac{[CH_2O^*]\cdot[O^*]}{[L]} \qquad (S5.42)$$

$$\frac{d[CO^*]}{dt} \cong 0 = k_9\frac{[CH_2O^*]\cdot[O^*]}{[L]} - k_{10}\frac{[CO^*]\cdot[O^*]}{[L]} \qquad (S5.43)$$

Therefore,

$$[CH_3CHO^*]\cdot[O^*] = \frac{k_7}{k_8}K_3P_{C_2H_4}[^*]\cdot K_1P_{O_2}[^*] \qquad (S5.44)$$

$$[CH_2O^*]\cdot[O^*] = \frac{2k_8}{k_9}[CH_3CHO^*]\cdot[O^*] = \frac{2k_7}{k_9}K_3P_{C_2H_4}[^*]\cdot K_1P_{O_2}[^*] \qquad (S5.45)$$

$$[CO^*]\cdot[O^*] = \frac{k_9}{k_{10}}[CH_2O^*]\cdot[O^*] = \frac{2k_7}{k_{10}}K_3P_{C_2H_4}[^*]\cdot K_1P_{O_2}[^*] \qquad (S5.46)$$

Substituting equations S5.44, S5.45, and S5.46 to S5.40 gives:

$$\frac{d[O^*]}{dt} \cong 0 = 2k_2K_1P_{O_2}[^*]^2 - 2k_{-2}[O^*]^2 + (k_6 - 4k_7)K_1P_{O_2}K_3P_{C_2H_4}[^*]^2 \qquad (S5.47)$$

Equation S5.47 can be transformed to:

$$2k_{-2}[O^*]^2 = K_1P_{O_2}\{2k_2 + K_3P_{C_2H_4}(k_6 - 4k_7)\}[^*]^2 \qquad (S5.48)$$

Solving equation S5.14 leads to [O*] = β[*], where β represents:

$$\beta = \sqrt{\frac{K_1P_{O_2}\left\{2k_2 + K_3P_{C_2H_4}(k_6 - 4k_7)\right\}}{2k_{-2}}} \qquad (S5.49)$$

Therefore, equations S5.44, S5.45, and S5.46 can be expressed as:

$$[CH_3CHO^*] = \frac{K_3P_{C_2H_4}(k_7K_1P_{O_2})}{k_8\beta} \qquad (S5.50)$$

$$[CH_2O^*] = \frac{2k_8}{k_9}[CH_3CHO^*] = \frac{2K_3P_{C_2H_4}(k_7K_1P_{O_2})}{k_9\beta} \qquad (S5.51)$$

$$[CO^*] = \frac{2k_8}{k_{10}}[CH_3CHO^*] = \frac{2K_3P_{C_2H_4}(k_7K_1P_{O_2})}{k_{10}\beta} \qquad (S5.52)$$

Then, we can rewrite the site balance (S5.1) as follows by defining [L] = α[*], where α represents:

$$\alpha = 1 + \beta + K_1P_{O_2} + K_3P_{C_2H_4} + \frac{K_3P_{C_2H_4}(k_7K_1P_{O_2})}{\beta}\left(\frac{1}{k_8} + \frac{2}{k_9} + \frac{2}{k_{10}}\right) \qquad (S5.53)$$

Finally, the analytical expressions of rates and χ can be written in the following forms:

$$\frac{r_{EO}}{[L]} = k_6\frac{[C_2H_4^*]\cdot[O_2^*]}{[L]^2} = \frac{K_3P_{C_2H_4}(k_6K_1P_{O_2})}{\alpha^2} \qquad (S5.54)$$

$$\frac{r_{CO_2}/2}{[L]} = k_{10}\frac{[CO^*]\cdot[O^*]}{2[L]^2} = \frac{K_3P_{C_2H_4}(k_7K_1P_{O_2})}{\alpha^2} \qquad (S5.55)$$

$$\chi = \frac{r_{EO}}{r_{CO_2}/2} = \frac{k_6}{k_7} \qquad (S5.56)$$

These expressions suggest that when ethylene is activated by only O_2^* to form EO or AA/CO_2, χ would be a constant that is equal to k_6/k_7, indicating that the selectivity would likely be determined by the barriers of reaction steps 6 and 7. This scenario agrees with the regime where we experimentally observed high coverages of O_2^{2-} at high P_{O2}/P_{C2H4} that showed a nearly constant value of χ (~0.8). DFT calculations suggest that higher value of χ in this regime could be due to the relatively lower barrier of ethylene activation on the O_2^{2-} containing surface.

References

1. Sanville, E.; Kenny, S. D.; Smith, R.; Henkelman, G., Improved grid-based algorithm for Bader charge allocation. *J. Comput. Chem.* **2007,** *28* (5), 899–908.
2. Henkelman, G. A.; Arnaldsson, A.; Jónsson, H., A fast and robust algorithm for Bader decomposition of charge density. *Comput. Mater. Sci.* **2006,** *36*, 354–360.
3. Linic, S.; Barteau, M. A., Control of Ethylene Epoxidation Selectivity by Surface Oxametallacycles. *J. Am. Chem. Soc.* **2003,** *125* (14), 4034–4035.
4. Linic, S.; Barteau, M. A., Formation of a Stable Surface Oxametallacycle that Produces Ethylene Oxide. *J. Am. Chem. Soc.* **2002,** *124* (2), 310–317.
5. Xu, Y.; Greeley, J.; Mavrikakis, M., Effect of Subsurface Oxygen on the Reactivity of the Ag(111) Surface. *J. Am. Chem. Soc.* **2005,** *127* (37), 12823–12827.
6. van den Hoek, P. J.; Baerends, E. J.; van Santen, R. A., Ethylene epoxidation on Ag(110): the role of subsurface oxygen. *J. Phys. Chem.* **1989,** *93* (17), 6469–6475.
7. van Hoof, A. J. F.; Hermans, E. A. R.; van Bavel, A. P.; Friedrich, H.; Hensen, E. J. M., Structure Sensitivity of Silver-Catalyzed Ethylene Epoxidation. *ACS Catal.* **2019,** *9* (11), 9829–9839.
8. Liu, J.-X.; Lu, S.; Ann, S.-B.; Linic, S., Mechanisms of Ethylene Epoxidation over Silver from Machine Learning-Accelerated First-Principles Modeling and Microkinetic Simulations. *ACS Catal.* **2023,** *13* (13), 8955–8962.
9. Özbek, M. O.; van Santen, R. A., The Mechanism of Ethylene Epoxidation Catalysis. *Catal. Lett.* **2013,** *143* (2), 131–141.
10. Ozbek, M. O.; Onal, I.; Van Santen, R. A., Ethylene epoxidation catalyzed by chlorine-promoted silver oxide. *Journal of Physics: Condensed Matter* **2011,** *23* (40), 404202.
11. Özbek, M. O.; Önal, I.; van Santen, R. A., Ethylene Epoxidation Catalyzed by Silver Oxide. *ChemCatChem* **2011,** *3* (1), 150–153.
